# The *Arabidopsis thaliana* *CONSTANS*-*LIKE 4* (*COL4*) – A Modulator of Flowering Time

**DOI:** 10.3389/fpls.2019.00651

**Published:** 2019-05-28

**Authors:** Yvonne Steinbach

**Affiliations:** Department of Plant and Microbial Biology, University of Zurich, Zurich, Switzerland

**Keywords:** plants, *Arabidopsis thaliana*, flowering time, *CONSTANS-LIKE 4* (*COL4*), repressor of flowering, *CONSTANS* (*CO*), *FLOWERING LOCUS T* (*FT*), *SUPPRESSOR OF OVEREXPRESSION OF CONSTANS 1* (*SOC1*)

## Abstract

Appropriate control of flowering time is crucial for crop yield and the reproductive success of plants. Flowering can be induced by a number of molecular pathways that respond to internal and external signals. In Arabidopsis, expression of the key florigenic signal *FLOWERING LOCUS T* (*FT*) is positively regulated by CONSTANS (CO) a BBX protein sharing high sequence similarity with 16 CO-like proteins. Within this study, we investigated the role of the Arabidopsis *CONSTANS*-*LIKE 4* (*COL4)*, whose role in flowering control was unknown. We demonstrate that, unlike CO, COL4 is a flowering repressor in long days (LD) and short days (SD) and acts on the expression of *FT* and *FT*-like genes as well as on *SUPPRESSOR OF OVEREXPRESSION OF CONSTANS 1* (*SOC1*). Reduction of *COL4* expression level leads to an increase of *FT* and *APETALA 1* (*AP1*) expression and to accelerated flowering, while the increase of *COL4* expression causes a flowering delay. Further, the observed co-localization of COL4 protein and CO in nuclear speckles supports the idea that the two act as an antagonistic pair of transcription factors. This interaction may serve the fine-tuning of flowering time control and other light dependent plant developmental processes.

## Introduction

Flowering is controlled by a network of well-established molecular pathways, which respond to internal and external signals, such as cold, light duration and quality, ambient temperature, as well as biotic and abiotic stresses (reviewed in: Andres and Coupland, 2012; Matsoukas et al., 2012; Leijten et al., 2018). In Arabidopsis, flowering is promoted mainly by the expression of the flowering integrators *FLOWERING LOCUS T* (*FT*), *SUPPRESSOR OF CONSTANS 1* (*SOC1/ AGL20*) and, redundantly to *FT*, it’s homolog *TWIN SISTER OF FT* (*TSF*) (Suarez-Lopez et al., 2001; An et al., 2004; Valverde et al., 2004; Yamaguchi et al., 2005). In Arabidopsis and other species, including tomato, tobacco, and rice, the FT protein (Corbesier et al., 2007) and FT-like protein ATC (Huang et al., 2012) are known to move from the leaf vascular tissue into the shoot apical meristem (SAM) mediated by FTIP1 (Liu et al., 2012). In the SAM, the formation of an FD-14-3-3 complex with FT or ATC and the other FT-homolog TERMINAL FLOWER 1 (TFL), induces or delays flowering, respectively, by changing the expression of the meristem identity genes *APETALA1* (*AP1*) and *CAULIFLOWER* (*CAL*) as well as *LEAFY* (*LFY*) and *AGAMOUS-LIKE 24* (*AGL24*) (Abe et al., 2005; Liu et al., 2008; Hanano and Goto, 2011; Taoka et al., 2011).

Central to the regulation of flowering is CONSTANS (CO), a nuclear zinc finger transcription factor belonging to the BBX protein family (Yanovsky and Kay, 2002, reviewed in Crocco and Botto, 2013; Gangappa and Botto, 2014). The CO protein acts in the vascular tissue of leaves, directly enhancing the expression of *FT* and *SOC1* under long-day conditions (Suarez-Lopez et al., 2001; Valverde et al., 2004; Corbesier et al., 2007). Thereby the flowering promotion by CO is dosage dependent. Reduction in CO level leads to late flowering in LD while overexpression promotes flowering in LD and SD (Putterill et al., 1995; Onouchi et al., 2000; Robson et al., 2001; Takada and Goto, 2003; Jang et al., 2008).

The *CO* expression and protein stability is regulated by the circadian clock and the photoperiod. In LD, accumulation of *CO* mRNA follows a diurnal cycle at the end of the light period, while in SDs *CO* mRNA level peaks during the night (Suarez-Lopez et al., 2001; Searle and Coupland, 2004; Valverde et al., 2004) due to the activity of photoreceptor complexes. Thereby expression of *CO* gets promoted by the blue light photoreceptor modul FKF1-GI (FLAVIN-BINDING KELCH REPEAT F-BOX 1; GIGANTEA) which degradates *CO*-repressors, such as CYCLING DOF FACTOR 1 (CDF1) (Imaizumi et al., 2005; Sawa et al., 2007; Demarsy and Fankhauser, 2009; Fornara et al., 2009; Song et al., 2012, 2014). In addition the blue light photoreceptors CRYPTOCHROME 1/2 (CRY1/CRY2) and the far-red light-absorbing phytochrome A (PHYA) control the CO protein stability by repression of the CONSTITUTIVE PHOTOMORPHOGENIC 1/SUPPRESSOR OF PHYA-105 (COP1/SPA) E3 ubiquitin ligase at the end of the day. While the red light-absorbing phytochrome B (PHYB) together with COP1/SPA targets CO to the proteasome in the morning and in the dark (Laubinger et al., 2004, 2006; Jang et al., 2008; Lazaro et al., 2012, 2015). Thus the circadian clock regulated transcription and the photoperiod dependent posttranslational control results in accumulation of CO-protein only in LD at dusk and remains low in SDs. Thereby the quantitative balance between the flowering activator CO and flowering repressors, such as the plant specific TEMPRANILLO (TEM1 and TEM2), is essential in the control of *FT* activation or repression, respectively (Castillejo and Pelaz, 2008; Osnato et al., 2012).

In general, the evolution of regulatory genes is an important aspect of diversification of developmental processes. *CO*-like genes are already present in moss (Zobell et al., 2005) or in green algae. The *Chlamydomonas reinhardtii* CO homolog *CrCO* strongly influences growth, cell shape and size, reinforces the circadian clock, and is involved in regulation of the starch synthase GRANULE BOUND STARCH SYNTHASE1 (GBSS1) (Serrano et al., 2009).

In Arabidopsis, 17 *CONSTANS-LIKE* (*COL*) genes were identified (Khanna et al., 2009) also referred as BBX1-BBX17. These genes have one or two conserved N-terminal B-Boxes for DNA-protein interaction and a conserved C-terminal CCT (CO, CO-like, TOC1) domain for protein-protein interaction resulting in nuclear localization. The COLs are structural classified into group 1, that have two functional B-Boxes (*CO*, *COL1-COL5*), group 2, that have one functional B-Box and a diverged zinc finger domain [*COL9*-*COL15*, (Cheng and Wang, 2005), and group 3, that have only one functional B-Box (*COL6-COL8*, *COL16*, Robson et al., 2001; Khanna et al., 2009; Wang et al., 2013]. The family of that B-box proteins also comprises additional BBX proteins (BBX18–BBX32) which do not contain the conserved CCT-domain (reviewed in Gangappa and Botto, 2014).

The biological function of the COLs is reported in a broad range of developmental processes: in flowering time, branching, shade avoidance response (SAR), photomorphogenesis or root elongation. Similar to CO, COL5 acts as accelerator of flowering (Hassidim et al., 2009). The COL3 and COL9 are reported to repress flowering in LD (Cheng and Wang, 2005; Datta et al., 2006). COL1 and COL2 are involved in fluence-rate dependent changes in circadian period, similar to what was seen for photoreceptors (Ledger et al., 2001). Both, COL3 and COL7 are involved in the red-far-red-light signaling regulating the SAR (Datta et al., 2006; Wang et al., 2013; Zhang et al., 2014). In addition, COL7 regulates branching of the shoot and was reported, similar to CO, to regulate the expression of the starch synthase GBSS (Ingkasuwan et al., 2012; Wang et al., 2013; Ortiz-Marchena et al., 2014). But so far, the functional importance of *COL4* has not been extensively investigated.

Here we demonstrate that Arabidopsis COL4 functions as transcriptional repressor of *FT* and therefore of flowering. A natural variation of the Arabidopsis *COL4*-gene, which results in a non-functional protein, was found in the Arabidopsis ecotypes Landsberg (La) and Landsberg erecta (Ler), but not in Columbia (Col) or Wassilewskaja (Ws). This occurrence might partially explain the differences in flowering time between these ecotypes. The results obtained within this study indicate that COL4 is needed for the fine-tuning of the expression of *FT* and less important also *ATC*, thus allowing full activity dependent of the photoperiodic pathway to induce flowering.

## Materials and Methods

### Plant Material

*Arabidopsis thaliana* seeds of ecotype Columbia (Col), Landsberg (La-0) and Landsberg-erecta (Ler) and T-DNA insertion lines were obtained from the NASC seed stock center. Individual T-DNA insertion alleles, indicated by dash-numbers, were confirmed by PCR. Lines used were *col4-1* (Salk_149180), *col4-2* (Salk_092012C), *col4-4* (Salk_102886), *col1-1* (Salk_024856C), *col3-1* (SALK_040211C, Datta et al., 2006), *col9-1* (SALK_137159, Cheng and Wang, 2005) and *co*-*10* (SAIL_24_H04). Seeds of *co-1*, *ft-10* and *soc1-2* have been described (Redei, 1962; Lee and Amasino, 1995; Lee et al., 2000; Yoo et al., 2005) and were kindly provided by D. Weigel (*ft-10*), I. Lee (*soc1-2*), B. Ayres (*co-1*). All mutant lines were in Columbia background except for *co-1* (La-0; Redei, 1962). Double mutants were identified among progeny of appropriate crosses by PCR with gene specific primers (Supplementary Table S4). The allele *col4*^La^ is a natural point mutation in La-0 and harbors a premature stop codon after amino acid position 31 (G + A nucleotide deletion at position 82+83 of the genomic sequence). The alleles *col4*^La^ and *co-*1^La^ as well as *COL4*^Col^ were backcrossed into Columbia or into La-0, respectively, resulting in at least three independent lines, indicated by dashed letters (*col4*^La^-A–D; COL4^Col^-A–E).

### Generation of COL4 Overexpression Lines

For overexpression analysis, the rps5a::gCOL4 plasmid was generated, carrying the full-length genomic sequence of *COL4* of Columbia in the modified binary destination vector pB7WG2 (Karimi et al., 2002; Erilova et al., 2009). Floral dip-transformation (Clough and Bent, 1998) of the plasmid into *col4*^La^ or *co-1* plants resulted in at least three independent transgenic plant lines (*col4*^La^ *COL4oe*-16, -19, -21, -23, -26, -27; *co*-1 *COL4oe*-1, -2, -8) selected by growth on plates containing BASTA.

### Growth Conditions and Flowering Time

Seeds were sterilized, stratified for 2–3 days at 4 °C, and plants were grown on plates on solid 0.5× Murashige and Skoog (MS) basal salt medium (Duchefa, Brussels, Belgium). Plants were analyzed on plates or transferred to soil (“Einheitserde,” Klasman, Switzerland) 10 days after germination. Alternatively, seeds were directly sown on soil. Plants were grown in Conviron growth chambers with mixed cold fluorescent and incandescent light as indicated for each experiment (80 or 130 μmol m^-2^ s^-1^, 21 ± 2°C) under long day (LD, 16 h/8 h day/night cycle) or short day (SD, 8 h/16 h day/night cycle) photoperiods. Flowering time was scored as described (Möller-Steinbach et al., 2010). Each presented flowering time analysis represents one out of at least two performed experiments.

### Phylogenetic Analysis

Conservation analysis of COL4 across the green lineage, was performed with sequences harvested from Phytozome 12 ^1^ and NCBI Blast search ^2^. Potential homologs were selected using a threshold of 20% sequence similarity, removing duplicate sequences or splice variants and prioritized sequences with motifs in the N-terminus, such as MASKL (AS at the position of 2nd potential translational start side of COL4) and CDSCDK (BBox1 of COL4). Sequence Alignment was done by using MEGA6^3^ with default parameters (pairwise alignment gap opening = 10.0, pairwise alignment gap extension = 0.1, multiple alignment gap opening = 10.0, multiple alignment gap extension = 0.2, and minimum gap separation distance of 4), further refined manually and used for construction of the phylogenetic tree in MEGA6 by the maximum likelihood method with 500 bootstrap replicates and the Jones-Taylor-Thornton (JTT) model.

### RNA Isolation and Quantitative RT-PCR (RT-qPCR)

Total RNA was extracted as previously described (Hennig et al., 2003; Leroy et al., 2007; Alexandre et al., 2009). For RT-PCR analysis, 1 μg RNA was treated with DNase I (Promega, Dübendorf, Switzerland) and transcribed into cDNA using RevertAid First Strand cDNA Synthesis Kit (Fermentas, Nunningen, Switzerland) according to manufacturer’s instructions. RT-qPCR analysis was performed with gene-specific primers (Supplementary Table S5) on three technical replicates with the TaqMan^®^ Gene expression assay (Fermentas, Lucerne, Switzerland) and the Universal Probe Library set (UPL) (Roche Diagnostics, Rotkreuz, Switzerland) according to the manufacturer’s instructions and results were analyzed in normalization to PP2A as described earlier (Exner et al., 2009). The presented data represents the mean of at least two experiments.

### Transient Expression of COL4 in *Nicotiana benthamiana* Leaves

To ectopically express proteins of full length COL4 (MDP-COL4) or truncated COL4 (MAS-COL4), as well as CO protein, the full length coding sequence or the truncated coding sequence, respectively, were inserted into the respective binary destination vectors pB7YWG (YFP) or pEarly102 (CFP-HA), resulting in the constructs MDP-COL4-CFP (35S::MDP-COL4-CFP), and MAS-COL4-YFP (35S::MAS-COL4-YFP), as well as CO-YFP (35S::CO-YFP). For subcellular localization analysis, Agrobacterium (GV3101) harboring the fusion protein constructs were grown in liquid LB-cultures under selective conditions at 28°C for 48 h, centrifuged at 3000 *g* for 10 min at RT and resuspend in 10 mM MgCl_2_ to an OD 600 of 0.8 to 1. Agrobacterium cells harboring the fusion protein constructs and Agrobacterium cells harboring a construct for the expression of the p19 silencing suppressor were mixed at equal ratio, infiltrated into the lower epidermis of *N. benthamiana* leaves and plants were incubated for 3–4 days before fluorescence imaging using a Zeiss LSM780 confocal microscope.

### Confocal Laser Scanning Microscopy (CLSM)

Sub-cellular localization studies were conducted using a 40× 1.1 numerical aperture water-immersion objective on a Zeiss LSM 780 confocal microscope equipped with an argon laser (Carl Zeiss GmbH, Jena, Germany). CFP fluorescence was excited with the 458 nm argon laser line and detected between 462 and 500 nm. YFP fluorescence was excited with the 514 nm argon laser line and detected between 518 and 557 nm. Auto-fluorescence of chloroplasts was excited with the 514 nm argon laser line and recorded between 662 and 721 nm. Image processing was done using ZEN 2011 software (Carl Zeiss GmbH, Jena, Germany) and ImageJ software^4^.

### Immunodetection

Tobacco leaf material was harvested 2–3 days after infiltration and ground in liquid nitrogen. Infiltrated leaf tissue and non-infiltrated leaf tissue (as control) was incubated in SDS-buffer (0.1 g / 1 ml buffer) for 20 min at 60°C. The PAGE run with 10 μl sample on a 10% SDS-Gel for 1 h at 20 mA. A wet-immunoblot was performed at 4°C at 100 V in 1 h on Nitrocellulose membrane activated by Blotting-buffer (20% EtOH, 25 mM Tris, 192 mM glycine). Anti-HA (1:5000, Roche) and anti-rabbit (1:10000, HRP coupled, Roche) were used to detect MDP-COL4-CFP. The anti-GFP (1:5000, rat, Jackson ImmunoResearch) and anti-rat (1:10000, HRP coupled, Roche) were used to detect MDP-COL4-CFP and MAS-COL4-YFP. Results were visualized by detection of chemiluminescence (ECL-detection kit, Roche) in a gel imager (Alpha Innotech).

## Results

### COL4 Is a Flowering Repressor Acting Antagonistically to CONSTANS

**FIGURE 1:**
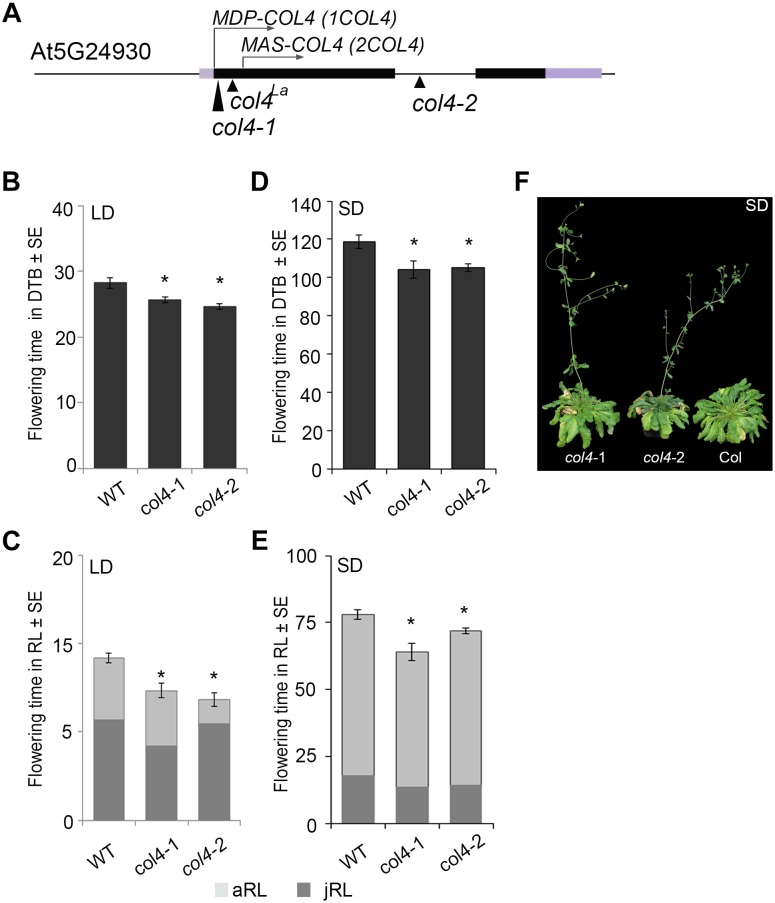
Characterization of *col4*-mutant alleles. **(A)** Scheme of the *COL4* gene (AT5G24930) including the translational start site for full length COL4 (MDP-COL4) and the potential truncated COL4 (MAS-COL4). Shown are as well the insertion site of the independent T-DNA lines *col4-1* (1st exon), *col4-2* (intron), and a point mutation line *col4*^La^ (1st exon) at the *COL4* locus. Black bars represent the two exons around the single intron, purple bars represent the 5′/ 3′UTRs, the arrows show the translational start site ATG1 (*MDP-COL4*) and the potential second translational start site ATG2 (*MAS-COL4*, without N-terminal peptide extension). **(B–E)** Flowering time of Columbia and the two independent T-DNA insertion alleles *col4-1* and *col4-2* in **(B,C)** long days (LD, 16 h light/8 h dark) and **(D,E)** short days (SD, 8 h light/16 h dark) measured in days to bolting (DTB) ± SE **(B,D)** and rosette leaf number at bolting (RL) ± SE **(C,E)** (*n* ≥ 14). Significance of difference was tested using *t*-tests. Asterisks denote differences that were significant at *p* < 0.05 to WT Columbia (^∗^). The graphs represent the results of one out of at least two independent experiments with the same outcome. **(F)** Phenotype of 115 days-old *col4*-1, *col4*-2, and WT Columbia plants grown in SD.

In Arabidopsis, the function of *CO* as a key floral activator is not conserved in other *COL* genes (see above). While some are also flowering activators, such as *COL5*, others are repressors of flowering, such as *COL3* and *COL9*. Here, we analyzed the flowering time of two independent *col4* mutants in the Columbia wild-type background. The *col4-1* allele carried a T-DNA insertion in the 1st exon, directly after the translational start site (Figure 1A). The *col4-2* allele carried a T-DNA insertion in the single intron of *COL4* (Figure 1A). Both were knockout alleles, since no full-length transcript was detected in these lines indicating the loss of full length COL4 protein, referred to as MDP-COL4. M-D-P represent the three first amino acids (aa) of the protein (Supplementary Figure S1). In the *col4*-*1* allele but not in *col4*-*2*, mRNA transcript of a truncated COL4 was observed, most likely referred to as MAS-COL4, starting with the 3 aa M-A-S. Both alleles disclosed a small but significant acceleration of flowering when days to bolting (DTB) and rosette leaf number (RL) at bolting were determined. In LD, both alleles flowered 2–3 ± 0.5 days earlier compared to wild-type (28 ± 0.8 DTB, Figure 1B). This effect was stronger in SD (Figure 1D), where both alleles flowered around 14 days earlier compared to wild-type (118 ± 3.4 DTB). In addition, the early flowering phenotype was reflected in number of RL. The *col4* alleles had less leaves at bolting compared to wild type in LD and SD (Figure 1C,E). Because both alleles disclose the same flowering behavior, the observed truncated transcript results rather in non-functional protein or in protein with different function than regulation of flowering. More important, the earlier flowering was rather a result from reduction of the juvenile and adult phase than growth defects, which can be seen in reduced number of juvenile (jRL) and adult leaves (aRL, Figure 1C,E). To corroborate our results, we also investigated the flowering time of other mutants of class group 1 *COL-*genes (all in the Columbia background) under our growth conditions. The selected mutants were reported in the literature as being early flowering (*col3-1*, *col9-1*), late flowering (*co*-1), or normal (*col1-1*). In our experiments, we could confirm these results, with the exception of *col1*-*1* (being in Col-background), which also flowered earlier (Supplementary Figures S2A,B). The phenotype of the *col4* alleles can be seen in Figure 1F. A third tested *col4* allele (*col4-4*) carried the T-DNA insertion 1000 bp upstream of the translational start site, which did not affect flowering time (Supplementary Figures S2E,F). However, we have strong evidence that *COL4* joins the group of *COL* genes encoding for flowering modulators, in the case of *COL4* as repressor of flowering.

### A Natural COL4 Mutation Is Worldwide Distributed in Arabidopsis Ecotypes

**FIGURE 2:**
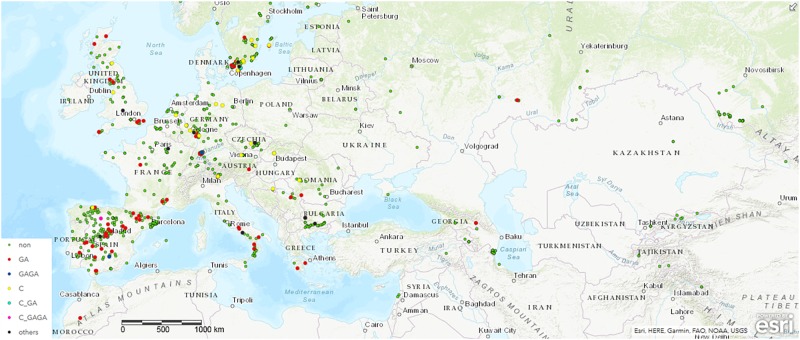
Distribution of ecotypes inheriting the natural variations in *COL4*. Shown are the origin of the ecotypes carrying (a) the common full length COL4-Sequence (green dots, non) coding for functional COL4 protein, referred here as MDP-COL4, (b) the GA-bp and GAGA-bp deletion at position 82–85 of the genomic sequence (red dots and blue dots, respectively), (c) the C-bp deletion at position 23 of the genomic sequence (yellow dots), (d) the two deletion events C-GA and C-GAGA (neon blue and pink, respectively). For the analysis, the genomic data from the 1001 Genomes project was used (The 1001 Genomes Consortium, 2016). Sequences/ecotypes used are listed in Supplementary Table S1. The map was generated by using longitude and latitude coordinates together with the web based ArcGIS software (http://www.arcgis.com/index.html). MDP and MAS – first three amino acids of the potential proteins. bp – basepair.

To monitor the role of COL4 on a natural and global range, we searched for potential *COL4* polymorphisms in Arabidopsis using the 1001 Genomes Dataset (The 1001 Genomes Consortium, 2016). Nearly 1000 worldwide distributed accessions contained the full length *COL4* sequence coding for a functional COL4 protein (MDP-COL4, Figure 2 and Supplementary Table S1A). Several accessions, however, disclosed natural variations in *COL4*, which caused premature stop-codons in the first exon of the sequence and most likely lead to loss of function of MDP-COL4 protein (Figure 2 and Supplementary Table S1B). Of these, nearly 80 accessions had lost a GA at pos. 82–83 of the genomic *COL4* sequence. Two accessions (IP-San-10 (Spain, ID9579) and Tä-KB-6 (Germany, ID9809)) had lost the 4 bp GAGA motif at pos. 82–85 of the genomic sequence. Nearly 30 accessions had lost a C at position 23 of the genomic sequence. The accessions KBG1-14 (Germany, ID9788) and Vår2-1 (Sweden, ID7516), as well as IP-Cdo-0 (Spain, ID9532) even displayed two deletions (C, pos. 23, GA, pos. 82–83, the latter: C, pos. 23, GAGA, pos. 82–85) causing a stop-codon at amino-acid-position 9. However, no clear correlation between the geographical distribution of the ecotypes and the occurrence of the natural *COL4* mutations could be detected (Figure 2 and Supplementary Figure S3). Only, the C-deletions seemed to occur mostly in ecotypes central and north to Europe, while the GA-deletions occurred mostly in ecotypes west and south to Europe. On a finer scale, the ecotypes containing the GA-deletions were mainly from Spain and Sweden, the GAGA- C-GAGA or C-GA-deletion were from Germany, Sweden and Spain, the C-deletion were mainly from Germany and Sweden, and the other nonsense mutations were from Bulgaria, France and Italy (Figure 2, Supplementary Figure S3, and Supplementary Tables S1A,B).

**FIGURE 3:**
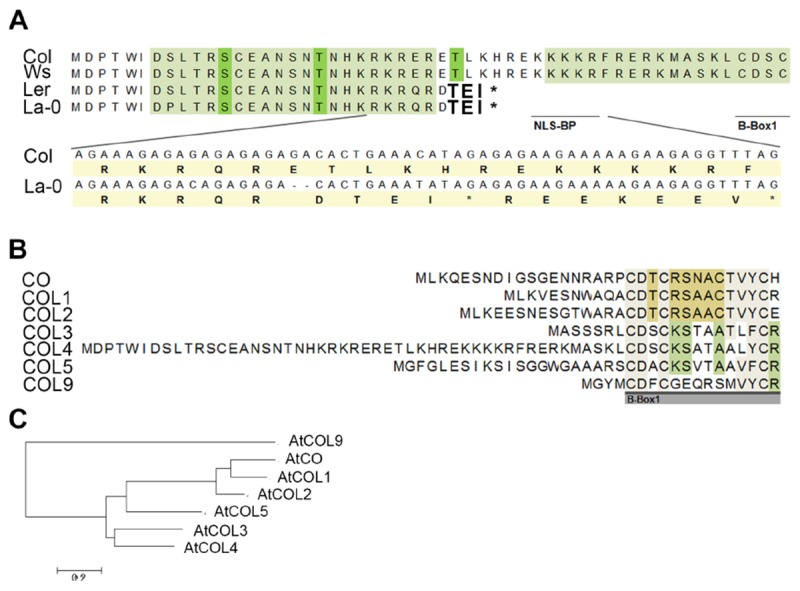
Protein sequence alignment of COL4. **(A)** Sequence alignment of COL4 from the common lab strains Col, Ws, Ler and La-0. A 2-bp-deletion caused a premature STOP codon in COL4 of Ler and La-0 [see insert in **(A)**]. Light green amino-acids (AA) represent phosphorylation hotspot and dark green AA a potential phosphorylation site. The potential bipartite nuclear localization signal (NLS-BP) and the 1st B-Box-start site are indicated by underlined AA. ^∗^STOP codon **(B)** Sequence alignment of the N-terminus of class 1 COL proteins (CO, COL1-5) and in addition of AtCOL9 conducted in Mega 6 (https://megasoftware.net/). Underlined gray bar = start of the B-Box1. **(C)** Phylogenetic analysis of group 1 COL proteins and in addition of AtCOL9.

However, subsequently, we sequenced the *COL4* gene from the most common laboratory strains Columbia (Col), Wassilewskaja (Ws), Landsberg (La), and Landsberg erecta (Ler), the latter three flower significantly earlier than Columbia in DTB and RL (Supplementary Figures S2C,D). In the accessions Col and Ws, the full length *COL4* sequence was detected, suggesting a functional COL4 protein (Figure 3A and Supplementary Figure S4A). In contrast, Ler and La-0 harbored the premature stop codon in the *COL4*-sequence at amino acid 32, due to the GA-bp-deletion at position 82–83 of the genomic sequence (Figure 3A and Supplementary Figure S4A). This *COL4* allele will hereafter be named *col4*^La^.

Interestingly, in sequence alignments the second occurring ATG (ATG2) in the open reading frame of *COL4* corresponded to the translational start site of COL3, the closest homolog of COL4 (Figure 3B,C). Hence, COL4 seems to contain an N-terminal extension which is unique to Arabidopsis COL4 within the group 1 COL proteins (Figure 3B). Within this N-terminal extension, several motifs were predicted by using MEME-suite ^5^; Supplementary Figure S4B), motifs such as LKXXE (present in many actin binding proteins, Howard et al., 1998), INSNTR, KRFRERK (containing a mono-partite NLS, Dingwall and Laskey, 1991), indicating that COL4 protein presents nuclear localization signals and protein binding sites at the N-terminus in addition to the ones at the C-terminus.

To define the uniqueness of the N-terminal extension of Arabidopsis COL4, a phylogenetic analysis was performed by extracting homologous COL4 protein sequences from Phytozome (see footnote 1) and the NCBI BLAST database^6^. Homologs of COL4 proteins could be identified across the green lineage, from Brassicaceae, Malvids, Malpighiales, Fabids, Rosids, Asterids, and monocots, as well as from mosses and green algae (Supplementary Table S2). Nevertheless, the mentioned N-terminal extension was unique to Arabidopsis COL4 within the homologous COL4 proteins (Supplementary Figure S5). Interestingly other long N-terminal extensions were found for homologs of Arabidopsis COL2 and COL5 in grape vine (*Vitis vinifera*), populus (*Populus trichocarpa*), strawberry (*Fragaria vesca*), switchgrass (*Panicum virgatum*) and citrus plants (*Citrus clementina*, Supplementary Figure S5, Supplementary Table S2).

### COL4 Expression Is Under Diurnal Control

**FIGURE 4:**
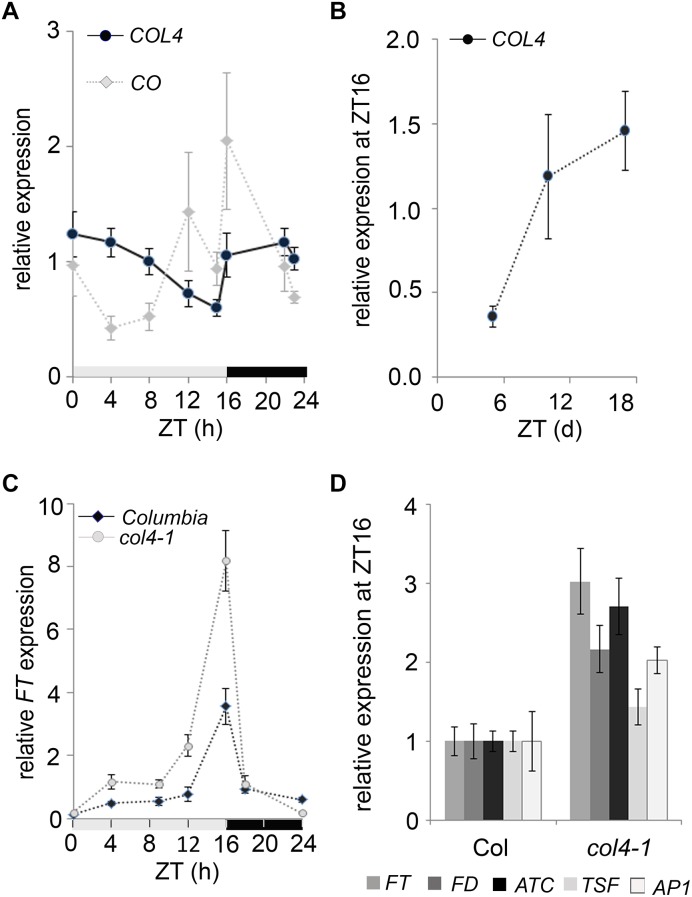
Expression kinetic of *COL4*. **(A)** Diurnal gene expression of *COL4* and *CO* in 6-days old wild type Columbia seedlings (*n* ≥ 20) grown in LD. Shown are relative expression values ± S.E. of quantitative RT-PCR (RT-qPCR) analysis. Light gray bar – light period; dark gray bar – dark period. **(B)** Kinetic of *COL4* expression in 5 to 18-days old wild-type Columbia seedlings grown in LD. Samples were harvested at ZT16. Shown are relative expression values ± S.E. (*n* ≥ 20 seedlings). **(C)** Diurnal *FT* expression in 6-days old seedlings of Columbia and *col4*-*1* mutant grown in LD. gray bar – light period, black bar – dark period. Shown are relative expression values ± S.E. (*n* ≥ 20 seedlings). Light gray bar – light period; dark gray bar – dark period. **(D)** Gene expression of *FT*, *ATC*, *TSF*, and *FD* and the direct FT target genes *AP1* at ZT16 in 10-days old seedlings of Columbia and *col4-1* grown in LD. Shown are relative expression values ± S.E. of RT-qPCR analysis (*n* ≥ 20 seedlings). The graphs represent the mean of at least two experiments.

To investigate the diurnal regulation of *COL4* expression, we carried out RT-qPCR-analysis with RNA harvested over a 24 h period from 6-days old wild type seedlings grown in LD conditions. Under these growth conditions, the *COL4* mRNA was preferentially expressed during the night, and mRNA levels decreased continuously during the day (Figure 4A). The expression of *CO* disclosed the expected peak toward the end of the day (ZT16) and was therefore anti-cyclically expressed to *COL4*. In addition, analysis of *COL4* expression in LD grown wild type Columbia seedlings aged between 5 and 18 days after germination disclosed a gradual increase with seedlings’ age (Figure 4B).

To further investigate the biological function of *COL4* in flowering time regulation, we tested the expression level of flowering time regulators in *col4-*1. The circadian expression rhythm of the flowering activator *FT* was not altered in *col4*-1. It still had its expression peak at ZT16 in LD (Figure 4C). Interestingly, however, its expression level was clearly increased in 6-days old *col4-*1 seedlings at ZT16 (Figure 4C). To further corroborate this result, *FT*, *FD*, *ATC*, and *TSF* expression were analyzed in 10 days-old seedlings of *col4-*1 and wild type (Figure 4D). We detected an up-regulation of the florigen *FT* and *FD* as well as milder up-regulation of the anti-florigen *ATC* at ZT16, whereas the expression of *TSF* remained as in wild type. The FT/ FD protein complex plays a strong role in flowering time initiation in LD by controlling the expression of *AP1* in the SAM. Consistently, we found an up-regulation of *AP1* expression in seedlings of *col4-1* mutant (Figure 4D), suggesting that the transition to flowering in *col4* occurred earlier due to higher FT and FD level and subsequently higher *AP1* expression. The increased expression of *FT* and *AP1* in the *col4* mutant was consistent with the observed decreased number of juvenile and adult leaves.

### COL4 Functions Upstream of the Flowering Time Activators FT and SOC1

**FIGURE 5:**
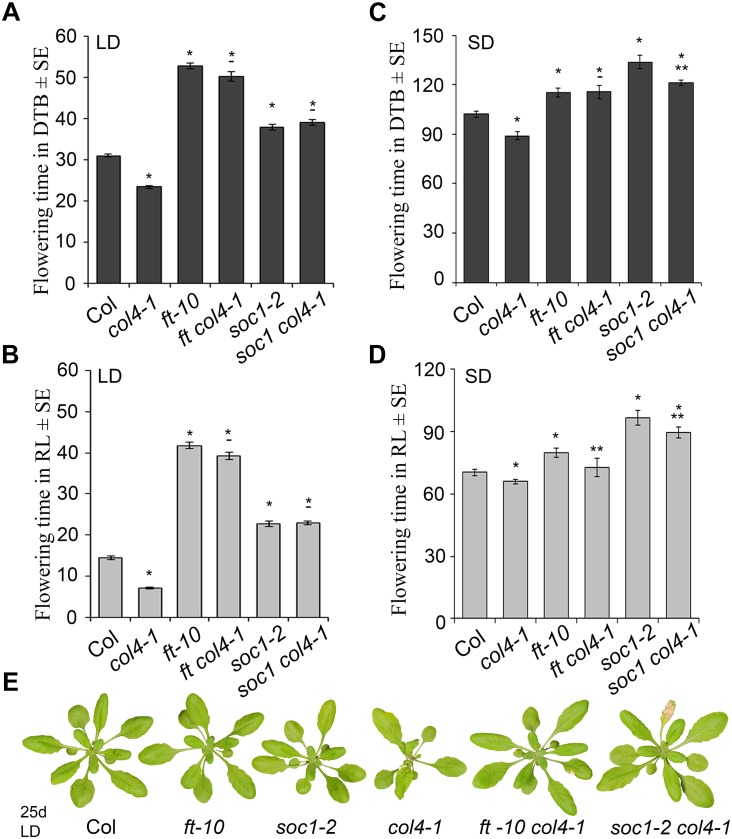
COL4 functions upstream of *FT* and *SOC1*. **(A–D)** Flowering time of the double mutants *ft-10 col4* and *soc1 col4* in LD **(A,B)** and SD **(C,D)** expressed in DTB and RL. Shown are mean values ± S.E. ^∗^ = *p*-value < 0.05 to Col, ^∗∗^ = *p*-value < 0.05 to corresponding late flowering single mutant. – = no significant difference to corresponding late flowering single mutant. **(E)** Phenotype of the single and double mutants grown for 25 days in LD, at the time point when *col4-1* flowered. All graphs represent the result of one out of at least two experiments with the same outcome.

To analyze the genetic interaction of *COL4*, *FT*, and *SOC1* (FT target), the flowering time of the double mutants *ft-10 col4-1* and *soc1-2 col4-1* in LD and SD was analyzed (Figure 5). In LD, *ft-10 col4-1* and *soc1-2 col4-1* flowered approximately at the same time with similar number of RL and DTB than the corresponding late flowering single mutant alleles *ft-10* or *soc1-2* (Figure 5A–B), suggesting an epistatic effect of *COL4* on *FT* and *SOC1*. The mutant phenotypes can be seen in Figure 5E. Therefore, in LD, *COL4* most likely acts in the same pathway than *FT* and *SOC1* and delays flowering by repression of *FT* and *SOC1* expression.

In SD, the double mutant *ft-10 col4-1* flowered at the same time than *ft-10* (Figure 5C,D). Compared to the situation in LD, where the wild-type flowered dramatically earlier than the mutants (Figure 5A), the delay of flowering of these mutants in SD was less pronounced (Figure 5C,D). Since *FT* is lowly expressed in SD, it is believed to have no significant impact on flowering. Hence, flowering is triggered through other SD-specific florigens, one of them is suggested to be *SOC1*. In our experiments, the *soc1-2 col4-1* double mutant flowered slightly but significantly earlier than *soc1-2* (121 DTB/90 RL and 134 DTB/97 RL, respectively, *p* < 0.05), and both flowered later than wild-type Columbia (102 DTB/70 RL) and the *ft-10* single mutant (115 DTB/80 RL, Figure 5C,D) suggesting an additive effect of *COL4* and *SOC1* on flowering in SD. Taken together, our results strongly suggest that *COL4* functions as a repressor of flowering upstream of *FT* and *SOC* in LD. In SD, *COL4* seems to contribute to flowering time in a *SOC1* independent way, most likely through repression of *FT* and *FT*-like genes, such as *ATC*.

### COL4 Expression Modifies Flowering Time

**FIGURE 6:**
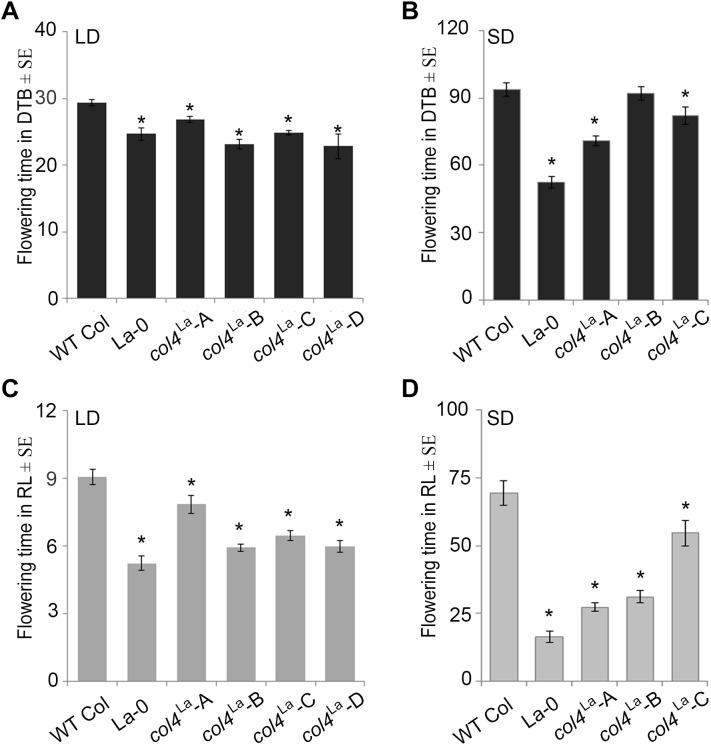
Loss of *COL4* accelerates flowering time. **(A–D)** Flowering time of independent Columbia *col4*^LA^ introgression lines (Landsberg allele *col4*^LA^ in Columbia background) in LD **(A,C)** and SD **(B,D)**; Shown are DTB (black bars) and RL (gray bars) at flowering ± SE (*n* ≥ 14 plants). Significance of difference was tested using *t*-tests. Asterisks denote differences that were significant at *p* < 0.05 to WT Columbia (^∗^).

Under our conditions, La-0 and L*er* flowered much earlier than Col in SD. To address the question if this was at least partly due to the altered COL4 gene in La-0 and Ler, the *COL4*^Col^ allele was introgressed into La-0, and this resulted in a delay in flowering compared to La-0 in LD and SD (Table 1). Thus, the introgression of *COL4*^Col^ could partially rescue the early flowering phenotype of La-0. In a reciprocal experiment, the *col4*^La^ allele was introgressed into Columbia, resulting in several independent Columbia *col4*^La^ plant lines all showing accelerated flowering in LD and SD (Figure 6A–D). These results strongly suggest that, COL4 protein was not functional as transcriptional repressor of flowering.

**Table 1:** Flowering time of *COL4* introgression lines in LD.

80 μE	DTB (LD)	RL (LD)
Col	29.39 ± 0.43	9.06 ± 0.34
*La-0*	24.69 ± 0.91*	5.23 ± 0.32*
*COL4^Col^-A (Col)*	29.46 ± 0.48+	10.31 ± 0.26*/+
*COL4^Col^-B (Col)*	34.44 ± 1.49*/+	11.89 ± 1.30*/+
*COL4^Col^-C (Col)*	25.73 ± 0.57*	7.73 ± 0.40*/+
*COL4^Col^-D-(Col)*	27.00 ± 0.52*/+	8.00 ± 0.38*/+
*COL4^Col^-E (Col)*	27.67 ± 0.84+	7.33 ± 0.60*/+


*Flowering time of independent *COL4* introgression lines (*n* ≥ 14) grown in LD under 80 μE light regime. Shown are days to bolting (DTB) and number of rosette leaves (RL) at flowering. Significance of difference was tested using *t*-tests. Asterisks denote differences that were significant at *p* < 0.05 to WT Col (^∗^), WT La-0 (^+^), single mutant (^∗∗^). Shown are mean values ± SE.*

**FIGURE 7:**
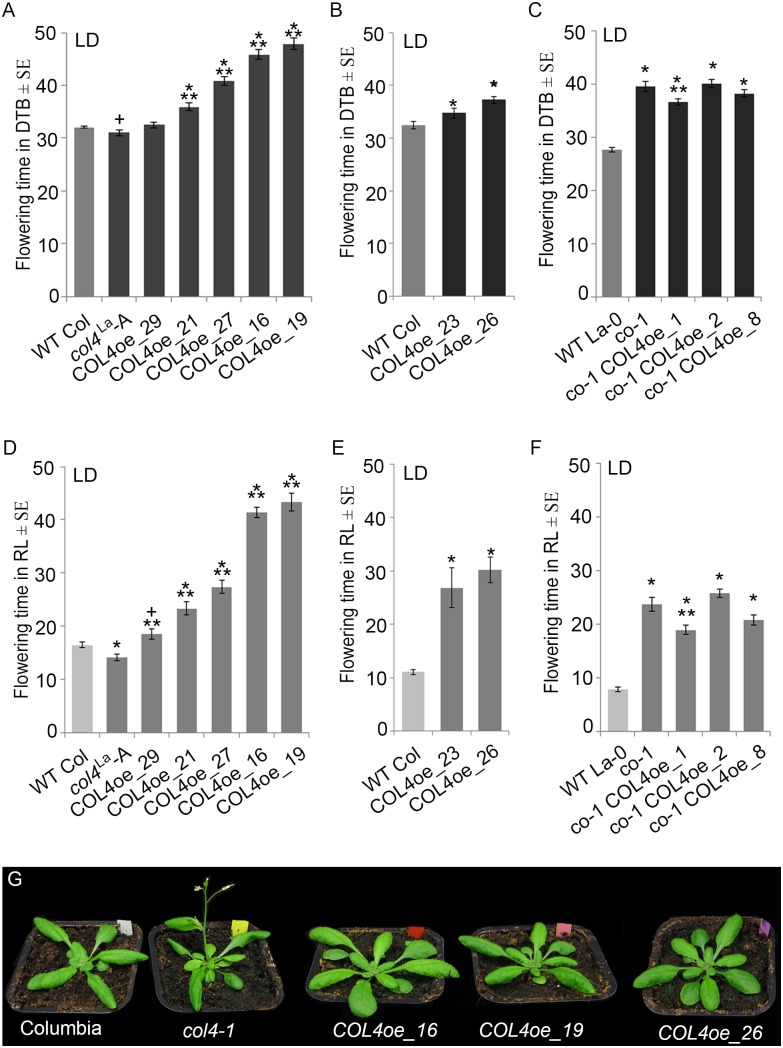
*COL4* overexpression delays flowering time. **(A–F)** Flowering time analysis in LD of independent transgenic lines overexpressing *COL4* in the *col4*^La^ Columbia allele (*COL4*-oe). The graphs show two experiments including the independent *COL4oe*-lines -16, -19, -21, -27, -29 **(A,D)** and the independent *COL4oe*-lines -23, -26 **(B,E)**. **(C,F)** Flowering time analysis of independent transgenic lines overexpressing *COL4* in the mutant *co*-1 (*co*-1 *COL4*-oe) in LD. Shown are DTB (black bars) and rosette leave numbers (RL, gray bars) at flowering ± SE (*n* ≥ 14 plants). Shown are mean values + S.E., p-value < 0.05 to WT (^∗^) or corresponding single mutant (^∗∗^), *p*-value < 0.1 to WT (+). **(G)** Plant phenotype of the single mutant and *COL4*oe lines grown in LD, at the time point when *col4-1* flowered.

We also overexpressed *COL4* under control of the rps5a-promoter (Erilova et al., 2009) in *col4*^La^ introgression lines (*COL4oe*, Figure 7). These plants showed a delay in flowering in LD and SD (Figure 7A,B,D,E and Supplementary Table S3A). *COL4*-overexpression in *co*-1 mutant (*co*-1 *COL4oe*) did not further contribute to a flowering delay (Figure 7C,F and Supplementary Table S3B). These results support the idea that COL4 fine-tunes regulation of flowering time by acting as a repressor of flowering.

**FIGURE 8:**
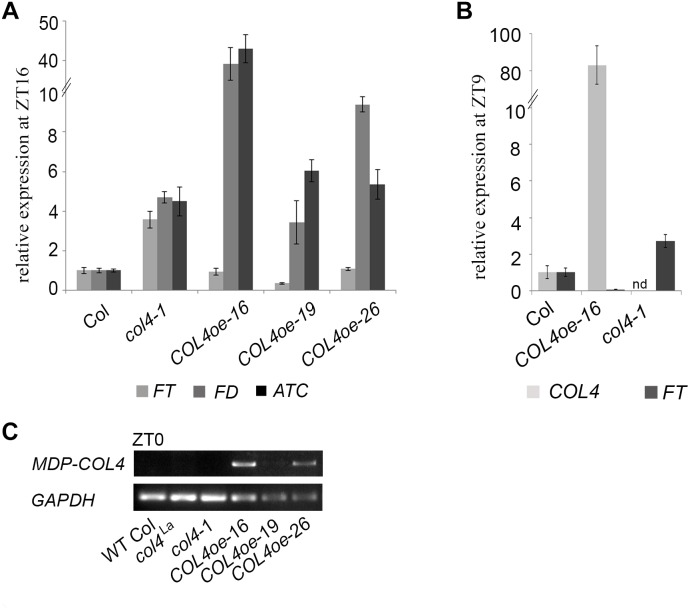
*COL4* overexpression modifies expression of key flowering time genes. **(A–B)** Gene expression of key flowering time genes in 24-days old Columbia, *col4* mutant plants and independent *COL4*-oe lines, grown in LD. Expression of the genes *FT*, *ATC*, and *FD* at ZT16 **(A)** and of *FT*, *COL4* after 9 h light period (ZT9) **(B)** was analyzed by using RT-qPCR. Five plants per independent line were pooled. Dash numbers represent individual transgenic *COL4* over-expression lines. nd – not determined. Shown are relative expression values ± S.E. All graphs represent the result of at least two experiments. **(C)** Gene expression of COL4 at the end of night (ZT0) in 24-days old Columbia, *col4* mutant plants and independent *COL4*-oe lines, grown in LD. Expression analysis was performed by semiquantitative PCR.

To further confirm our hypothesis, the expression of flowering time genes in *COL4oe* plants grown in LD was analyzed. We detected decreased *FT* expression in the *COL4*oe-lines, compared to *col4-1* mutant (Figure 8A), but expression of *ATC* and *FD* remained high at ZT16. We also detected a second peak of *FT* in the middle of the light period in Figure 4C, 8B. Nevertheless, *COL4* overexpression (Figure 8C) caused a shift in the *FT/ATC* balance toward *ATC* (Table 2). The change in expression and corresponding ratios between *FT*, *FD*, and *ATC* might explain the accelerated flowering of *col4*-mutants and the strong late flowering of *COL4*-oe lines in LD.

**Table 2:** Ratio of expression between FT/ATC, ATC/ FD and FT/FD.

LD	ratio	FT/ATC	ATC/FD	FT/FD
(A) LD	Col	1.46	33.14	48.43
	*col4-1*	2.68	15.72	42.07
(B) LD	Col	3.70	3.07	11.38
	*col4-1*	3.11	2.78	8.65
	*col4^La^*	5.49	0.56	3.09
	*COL4oe-16*	0.20	2.69	0.54
	*COL4oe-19*	0.23	5.09	1.18
	*COL4oe-26*	0.87	1.49	1.31


***(A–B)** Ratio of expression values obtained by RT-qPCR analysis of *FT*, *ATC* and *FD* in 10-days old seedlings **(A)** and in 24-day old RLs **(B)** grown in LD.*

### COL4 Co-localizes With CONSTANS in Nuclear Speckles

**FIGURE 9:**
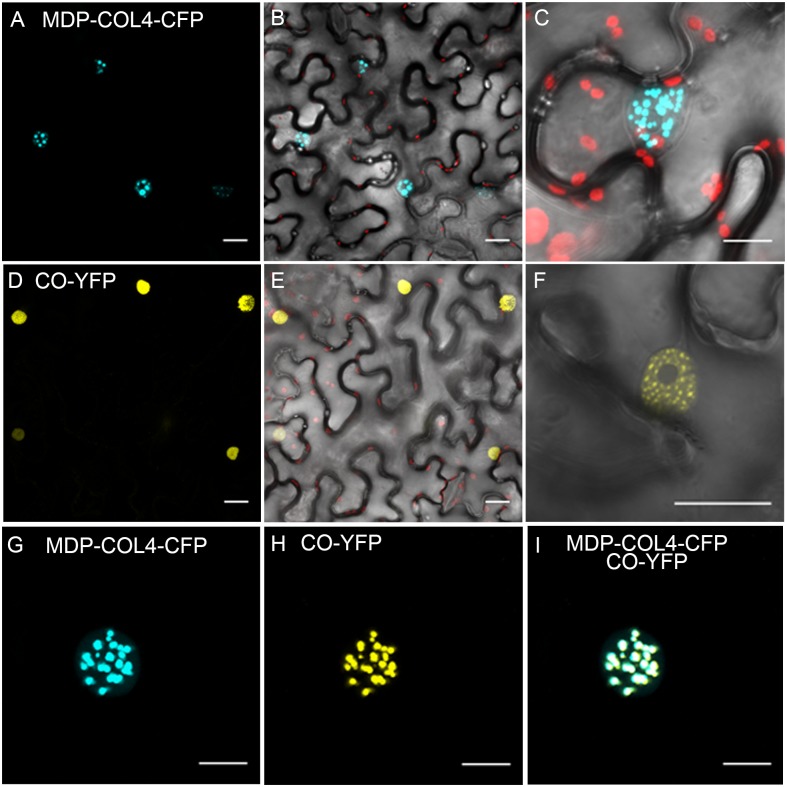
Co-localization of COL4 and CO in the nucleus. **(A–C)** MDP-COL4-CFP localization. **(A)** MDP-COL4-CFP fluorescence (blue) in the nuclei. **(B)** Merge of **(A)**, chlorophyll auto-fluorescence (red) and bright-field. **(C)** Zoom to one nucleus: Merge of MDP-COL4-CFP fluorescence, chlorophyll and bright-field. **(D–F)** CO-YFP localization. **(D)** CO-YFP fluorescence (yellow) in the nuclei **(E)** Merge of **(D)**, chlorophyll auto-fluorescence (red) and bright-field. **(F)** Zoom to one nucleus: Merge of CO-YFP fluorescence and bright-field. **(G–I)** The co-localization of MDP-COL4-CFP and CO-YFP in the nucleus: MDP-COL4-CFP fluorescence **(G)**, CO-YFP fluorescence **(H)** and **(I)** merge of **(G)** and **(H)**. Scale bars: 20 μm. Shown are images of tobacco leaf cells transiently expressing the respective fusion proteins.

To confirm that, similarly to CO, COL4 is localized to the nucleus and thus likely functions as transcription factor, a fusion between the full length *COL4* and the gene encoding for *CYAN FLUORESCENT PROTEIN* (*CFP*) was constructed under the control of the 35S-CaMV promoter (35S::MDP-COL4-CFP). A transient expression assay in tobacco leaf cells was used to assess the cellular localization of the MDP-COL4-CFP protein. As expected, the fluorescence of MDP-COL4-CFP was detected predominantly in the nuclei (Figure 9A–C). Additionally, to assess the co-localization of COL4 and CO, the yellow fluorescent fusion protein CO-YFP was transiently expressed under the control of the 35S-CaMV promoter in *N. benthamiana* leaves together with MDP-COL4-CFP (Figure 9D–F). Both, CO-YFP and MDP-COL4-CFP co-localized solely in the nuclei in nuclear speckles (Figure 9G–I), which might be cajal bodies (Datta et al., 2006).

**FIGURE 10:**
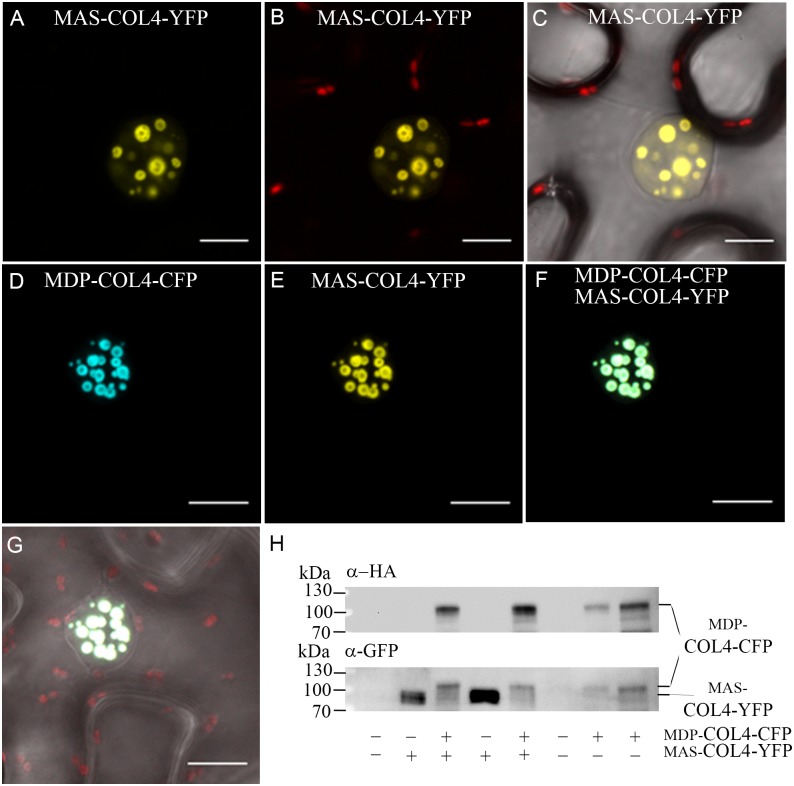
Co-localization of MDP-COL4 and MAS-COL4 in the nucleus. **(A–D)** Co-localization of MDP-COL4-CFP and MAS-COL4-YFP in the nucleus. **(A)** MAS-COL4-YFP fluorescence (yellow) in the nuclei. **(B)** Merge of **(A)** and chlorophyll auto-fluorescence. **(C)** Merge of **(A)**, chlorophyll auto-fluorescence (red) and bright-field. **(D)** MDP-COL4-CFP fluorescence (blue). **(E)** MAS-COL4-YFP fluorescence in the same nucleus as **(D)**. **(F)** Merge of **(D)** and **(E)**. **(G)** Merge of **(F)**, chlorophyll auto-fluorescence (red) and bright-field. Shown are images of tobacco leaf cells transiently expressing the respective fusion proteins. Scale bars: 20 μm. **(H)** Immunoblot analysis of transiently expressed COL4 proteins in *N. benthamiana* leaves. MDP-COL4-CFP carried the full length COL4, a HA- and CFP fusion tag. MAS-COL4-YFP carried the truncated COL4 from the 2nd translational start site (ATG2) and a YFP fusion tag.

Additional, the natural *MDP-COL4* transcript was detectable only at low level, but testing for *MAS-COL4* transcript resulted in a strong expression signal (Supplementary Figure S1). The question arose about the importance of the N-terminal extension and if a potential truncated protein would be synthesized and stable localized to the nucleus. Therefore, the localization of the two potential COL4 variants was evaluated. MDP-COL4-CFP and MAS-COL4-YFP were transiently expressed in *N. benthamiana* leaves, either co-expressed or in a single expression event. The transient synthesis of the proteins MDP-COL4-CFP and MAS-COL4-YFP could be visualized by excitation of the CFP- and YFP fluorescent tags at different wave lengths, whereby both co-localized solely in the nuclei in nuclear speckles (Figure 10A–G). In addition, the synthesis of both COL4 protein variants was evaluated by immunoblot analysis using different antibodies (Figure 10H; anti-HA for MDP-COL4-CFP; anti-GFP for MDP-COL4-CFP/MAS-COL4-YFP). Due to the long N-terminal extension, MDP-COL4 had a higher molecular weight than MAS-COL4-YFP, resulting in a band shift seen in Figure 10H. In general, COL4 protein, even truncated, seemed to nuclear co-localizes with the CO protein. But the function of the N-terminal extension and the potential truncated COL4 protein needs to be investigated in the future.

However, the full length COL4 localized to the nucleus into speckles and functioned as transcriptional repressor on the key flowering activator *FT* explaining the early flowering phenotype of *col4* mutants and the delay in flowering of *COL4*-overexpressing plants.

## Discussion

For plants the exact regulation of flowering time is critical for their reproductive success, whereby CO acts as a key activator of flowering in Arabidopsis and other plant species. To date, less is known about the biological function of its sequence homolog COL4. Only its role in salt stress response was reported (Min et al., 2014). In this study, we could disclose that the Arabidopsis COL4 functions as flowering repressor in LD and SD. Plants lacking functional COL4 displayed accelerated flowering, while COL4 overexpression delayed it. Interestingly, COL4 and CO, both being nuclear localized, adversely regulate the expression of common target genes, such as the florigen *FT*, its antagonist *ATC*, as well as *FD* or *SOC1*, and subsequently of the meristem identity gene *AP1*. In plants, FT and ATC or TFL1 proteins compete for forming a complex with FD. While the FT/FD interaction promotes flowering (Abe et al., 2005; Wigge et al., 2005; Taoka et al., 2011), ATC/FD form a flowering inhibitory complex (Hanano and Goto, 2011; Huang et al., 2012). In the *col4* mutants used in this study, *FT* and *FD* expression was upregulated relative to *ATC*, and the *ATC/FD* ratio was lower than in wild type. The changed balance of these target genes is the most likely reason for the observed earlier flowering phenotype of *col4*. Thus, antagonistically to CO, COL4 seems to fine-tune the balanced expression of these target genes to achieve optimal flowering time.

Several lines of evidence suggest that the balanced activity of repressors and activators of flowering can also have other, flowering-independent, functions. Some of these functionally antagonistic pairs seem to play roles as general growth regulators, as was discussed for the tomato FT-homolog and TFL1-ortholog (Lifschitz et al., 2006; Shalit et al., 2009; Krieger et al., 2010). Since COL4 was recently found to be involved in salt stress regulation (Min et al., 2014), BROTHER OF FT AND TFL1 (BFT), which was reported to function in a FD-BFT repressor complex under salt stress (Ryu et al., 2014), may be another FT-like gene under the control of the COL4/CO antagonistic pair. This hypothesis, however, still requires experimental evidence. The idea of a more general role of COL4 and COL homologs in plant development gets supported by the findings of our phylogenetic analysis as well as by others (Lagercrantz and Axelsson, 2000; Zobell et al., 2005; Serrano et al., 2009; Gangappa and Botto, 2014). We found sequence homologs of COL4 already in the mosses (*Physcomitrella patens*) (Griffiths et al., 2003; Shimizu et al., 2004; Zobell et al., 2005) or in green algae (Serrano et al., 2009). This indicates that COL4 has important functions in plants besides flowering control, similar to other COLs and BBX family members. Recent publications discuss a linkage between COLs and BBX proteins being involved in plant developmental processes spanning over transition to flowering, seedling de-etiolation, photomorphogenesis, responses to antibiotic stress and shade avoidance, plant architecture, e.g., branching, circadian clock regulations and hormone signaling (Ledger et al., 2001; Cheng and Wang, 2005; Datta et al., 2006; Hassidim et al., 2009; Wang et al., 2013; Wang and Dehesh, 2015; Graeff et al., 2016; Tripathi et al., 2016, for review see: Vaishak et al., 2019). The COLs, especially CO, form heterodimers with other BBX proteins while being mediated into larger protein complexes and inactivated that subsequently alters the flowering habit. For example, BBX30/BBX31, known as micro-proteins MiP1a/b, traps CO into TOPLESS complex (TPL) (Graeff et al., 2016), BBX19 (DBB1b) or BBX10 (COL12) bind CO and deplete its capacity of activating *FT* expression (Wang et al., 2014, 2015; Ordoñez-Herrera et al., 2018). Recently it was also shown that COL3, the closest COL4 homolog, interacts with BBX32 (EIP6) to mediate proper flowering, while BBX32 effects the circadian clock and is involved in photomorphogenesis as well (Park et al., 2011; Tripathi et al., 2016). Most of those interactions might be driven by the affinity of the BBox-domains. Due to COL4’s sequence similarity to COL3 and the COLs, COL4 might form such heterodimers and might have similar binding partners in a network of fine-tuning activity or inactivity, including the interaction with BBX32 in regulation of flowering time or with BBX micro-proteins in modulation of the circadian clock. This hypothesis, however, still requires experimental evidence.

Sequence variation matters for the choice of interaction partners. The frequent occurrence of genetic variation in the COL4 sequence, results in two groups of polymorphism, one present in northern Europe and the other in southern Europe. Within in those ecotypes, including the ecotype Landsberg, premature stop-codons in the first exon indicate non-functional full length COL4 but still might lead to the formation of a shorter COL4 protein. Potential dual translation of COL4 transcript was discussed already by Liu et al. (2013). The full-length version of COL4 contains a long N-terminal extension unique to COL4 within the group 1 COLs from Arabidopsis. This N-terminal peptide contains potential phosphorylation sites (S19 and T29), motifs for protein interaction (Howard et al., 1998), as well as additional NLS sequences (NLS-mono-partite: K-(K/R) × (K/R), Lange et al., 2007; NLS-bi-partite: (K/R)(K/R) × 10–12(K/R), Dingwall and Laskey, 1991), loss of which, however, did not prevent nuclear localization but it’s transcriptional role in flowering time. Nevertheless, these protein sequences might contribute to a specific regulation of COL4, which is necessary for its function in flowering time. Such extensions were also detected in homologs of AtCOL2 and AtCOL5 in vine, poplar, wild strawberry, switchgrass and citrus plants. It will be interesting to investigate the evolution of these long N-terminal extensions in correlation with additional functions for COL4 or other COL variants.

Transcription of *COL4* is under diurnal regulation and peaks during the night, anticyclical to *CO*. Further experiments will be necessary to determine whether and how COL4 protein stability is regulated. However, the high similarity of their protein sequences suggests a similar protein regulatory system for COL4 and CO. Both proteins are targeted to the nucleus (this study and Min et al., 2014), presumably due to their N-terminal B-Boxes, as was shown for AtCOL3 (Datta et al., 2006). Moreover, COL4 and all other COLs contain the VP-AS pair in the CCT-domain essential to interact with COP1/SPA1 (Ordoñez-Herrera et al., 2018), subsequently being ubiquitinated (Manzano et al., 2008; Brandao et al., 2009) and targeted to the proteasome during the night (Suarez-Lopez et al., 2001; Searle and Coupland, 2004; Corbesier and Coupland, 2006; Datta et al., 2006). In addition, the CCT-domain of COL4 interacts with the LKP family (ZTL, FKL), which was seen for the whole group 1 COLs in general but not COL7 or COL9 (Fukamatsu et al., 2005). ZTL affect the circadian clock being part of the SCF complex. Together with GI, ZTL degrades the circadian clock components of the PRR/TOC1 family (Más et al., 2003; Kim et al., 2010). Therefore it is most likely that COL4 protein synthesis and stability is tightly regulated by components of the circadian clock, such as TOC1/PRR or components of the proteasome, such as ZTL or LKP2 in the process of circadian gating of light. The location of this regulation might be cajal bodies. Besides TOC1, CO co-localizes with the whole LKP-family in nuclear cajal bodies (Strayer et al., 2000; Gall, 2000, 2003). These spherical structures are frequently associated with the nucleolus and might regulate assembly of the so-called complexes for transcription (Fukamatsu et al., 2005). As both, COL4 and CO co-localize in the nucleus, those observed intense accumulation “blobs” are most likely these cajal bodies. To fit all the observations into a more refined model for flowering control of COL4, further investigations will be necessary.

Summarizing, based on our results, COL4 is a transcriptional repressor of flowering in LD and SD. In addition, COL4 most likely has similar BBX interaction partners than CO, regulating plant developmental processes including flowering time. Further COL4 most likely underlies similar mechanisms in regulation of protein stability than CO. It is tempting to speculate that COL4 and CO function as an antagonistic pair in flowering time regulations, similarly to what was discussed for FT and TSF or ATC (Abe et al., 2005; Wigge et al., 2005; Hanano and Goto, 2011). As a conceptual model it might be interesting to note that FT and TFL1 or ATC are also homologs that favor or repress flowering. CO and COL4 might be another such pair. Flowering time control could be more delicate with these antagonistic pairs compared to the simpler situation with only one activator. The main point of this additional control may be to prevent leakiness in the absence of an activator.

## Author Contributions

YS conceived and carried out the experiments, analyzed the data, and wrote the manuscript.

## Conflict of Interest Statement

The author declares that the research was conducted in the absence of any commercial or financial relationships that could be construed as a potential conflict of interest.

## Footnotes

1 www.phytozome.net

2 www.ncbi.org

3 http://www.megasoftware.net/

4 http://rsbweb.nih.gov/ij/

5 meme-suite.org

6 https://www.ncbi.nlm.nih.gov/
