# Supplementary material for: The Arabidopsis thaliana CONSTANS-LIKE 4 (COL4) – A Modulator of Flowering Time
Source: Front Plant Sci. 2019 May 28;10:651. doi: 10.3389/fpls.2019.00651 (PMC6546890; doi:10.3389/fpls.2019.00651)
Supplement: Supplementary file 1 [file Data_Sheet_1.PDF]

Supplementary Material

The *Arabidopsis thaliana CONSTANS-LIKE 4* (*COL4*) – a modulator of flowering time

Yvonne Steinbach[1*]

*** Correspondence:** Yvonne Steinbach: yvonne.steinbach@uzh.ch

Supplementary Figures

Fig.S 1: Expression phenotype of the knockout alleles *col4-1* and *col4-2*

Fig.S 2: Flowering time of mutants in COL1, COL3 and COL9 and the common ecotypes Col, Ws, Ler, Enk, C24 grown in LD

Fig.S 3: Worldwide location of ecotypes inheriting the natural COL4 polymorphisms.

Fig.S 4: AtCOL4 Protein Sequence alignment of common ecotypes.

Fig.S 5: Molecular Phylogenetic analysis of COL4 like genes in green plant lineage.

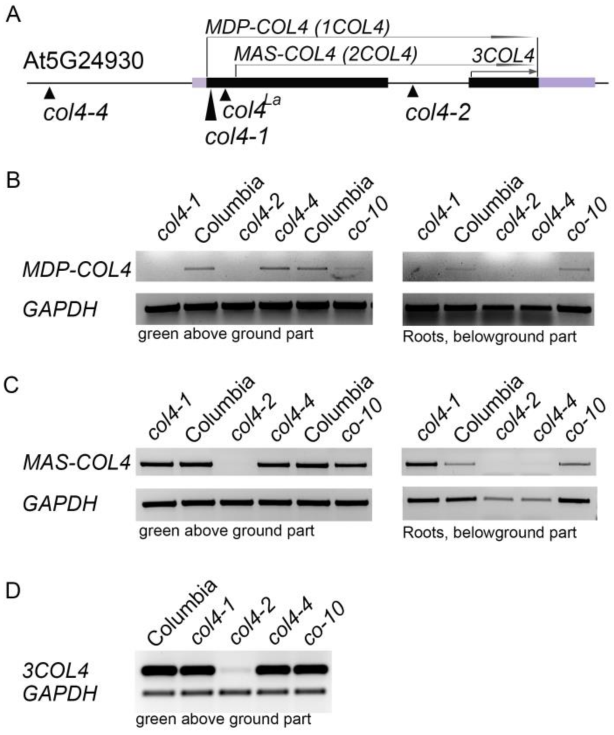

Fig.S 1: Expression phenotype of the knockout alleles *col4-1* and *col4-2*

(A) Scheme of the *COL4* gene (AT5G24930) including the translational start site for full length COL4 (MDP-COL4) and the potential truncated COL4 (MAS-COL4). Shown are the insertion site of the independent T-DNA lines *col4-1* (1st exon), *col4-2* (intron), and a third T-DNA-line *col4-4* (promoter region) at the *COL4* locus. Black bars represent the two exons around the single intron, purple bars represent the 5`/ 3`UTRs. The arrows show the region of the generated PCR fragments for semi-quantitative analysis of *COL4* expression. (B-D) Semi-quantitative RT-PCR of expression of *COL4* in the green seedlings part (cotelydons, SAM, hypocotyl, left panel) and the roots (right panel) of 10d old seedlings grown in 12h/12h light/dark regime: (A) RT-PCR of full length *COL4* transcript (*MDP-COL4*). Both mutants, *col4-1* and *col4-2* are knockout for full length *MDP-COL4* - no transcript of *COL4* could be detected. But the natural expression of *MDP-COL4* occurred at an extreme low level in seedlings of Columbia, *col4-4* and *co-10.* (B) RT-PCR of truncated *COL4* PCR fragment (*MAS-COL4*) spanning the region from the second ATG of the reading frame to the stop codon of *COL4* mRNA. The *col4-2* allele was knockout for the *MAS-COL4*, while transcript could be measured in *col4-1* indicating a read out from the T-DNA insertion most likely resulting in non-functional protein. Interestingly, the natural expression of *MAS-COL4* occurred at a stronger level compared to the *MDP-COL4* in Columbia, *col4-4* and *co-10* which lead to the question of different functions of COL4 protein than flowering time regulation. Dashed numbers indicate independent T-DNA insertion lines.

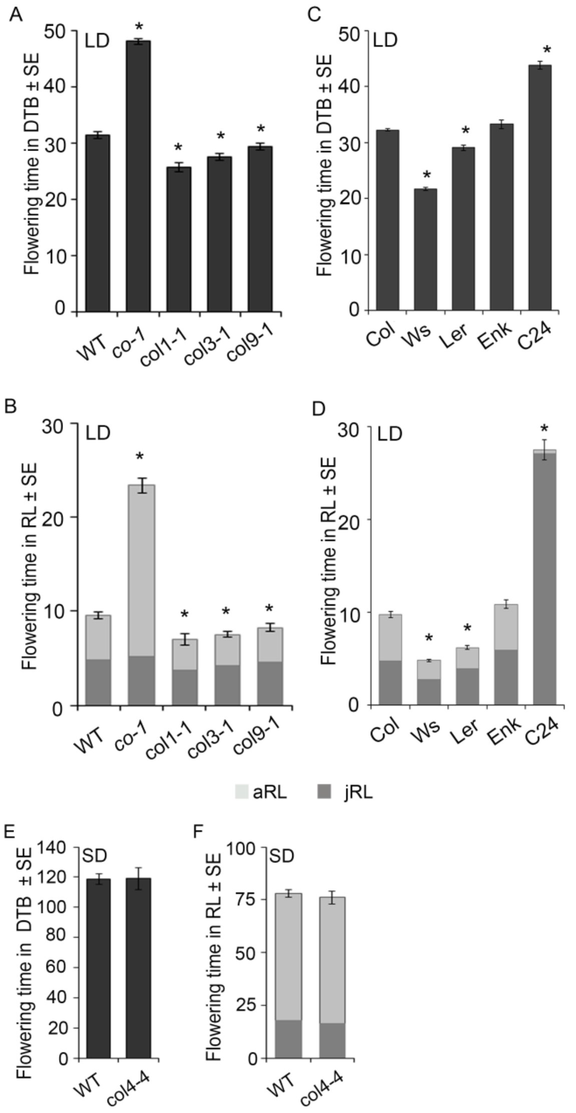

Fig.S 2: Flowering time of Arabidopsis plants, mutants in *COL* genes and certain ecotypes

(A-F) Flowering time of mutants in *COL4* genes and specific ecotypes. Shown are days to bold (DTB, black bars) at flowering ± SE and total rosette leave numbers (RL) at flowering ± SE. The graphs containing the grey bars are subdivided into adult rosette leaves (aRL, light gray bars) and juvenile rosette leaves (jRL, grey bars). (A-B) Mutants in class 1 COL-genes, *co-1*, *col1-1*, *col3-1* and *col9-1* grown in LD. Shown are DTB ± SE (A) and RL ± SE (B) at flowering. All mutants were in Columbia background. Dashed numbers indicate independent T-DNA insertion lines. (C-D) Common laboratory strains Columbia, Ws, Ler, Enk and C24 grown in LD. Shown are DTB ± SE (C) and RL ± SE (D) at flowering. (E-F) Flowering time of the mutant allele *col4-4*, containing a T-DNA insertion in the promoter region, grown in SD. Shown are DTB ± SE (A) and RL ± SE (B) at flowering. The insertion into the promoter region does not change the flowering phenotype. Significance of difference was tested using t-tests. Asterisks denote differences that were significant at $p < 0.05$ to WT Columbia (*). The graphs represent the results of one out of at least two independent experiments with the same outcome.

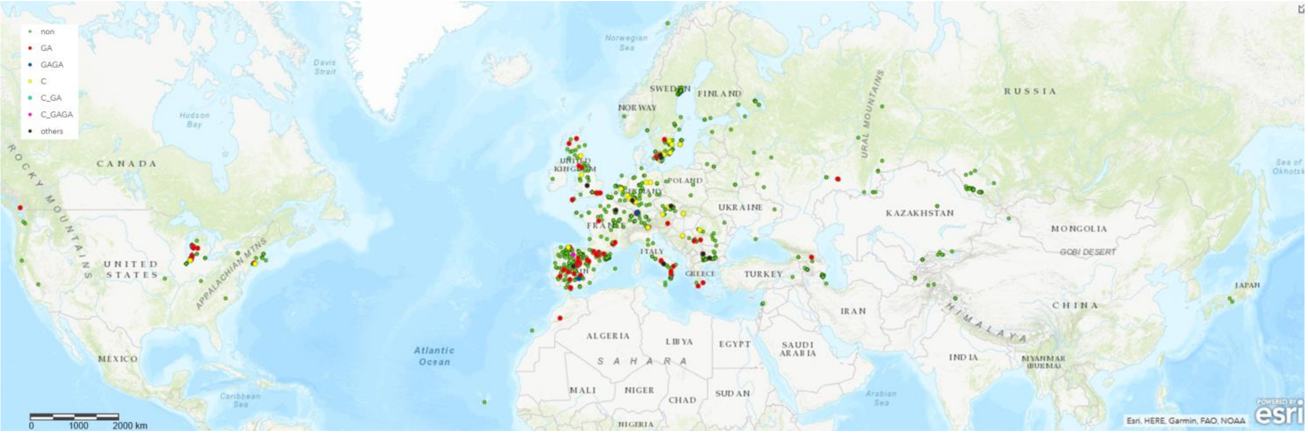

Fig.S 3: Worldwide location of ecotypes inheriting the natural COL4 polymorphisms.

Shown are the origin of the ecotypes carrying a) the common full length COL4-Sequence (green dots) coding for functional COL4 protein, referred here as MDP-COL4, b) the GA-bp and GAGA-bp deletion at position 82-85 of the genomic sequence (red dots and blue dots, respectively), c) the C-bp deletion at position 23 of the genomic sequence (yellow dots), d) the two deletion events C-GA and C-GAGA (neonblue and pink, respectively). For the analysis, the genomic data from the 1001 Genomes project was used (The 1001 Genomes Consortium, 2016). Sequences/ecotypes used are listed in Table S 1. The map was generated by using longitude and latitude coordinates together with the web-based ArcGIS software (http://www.arcgis.com/index.html). MDP and MAS – first three amino acids of the proteins. bp – basepair

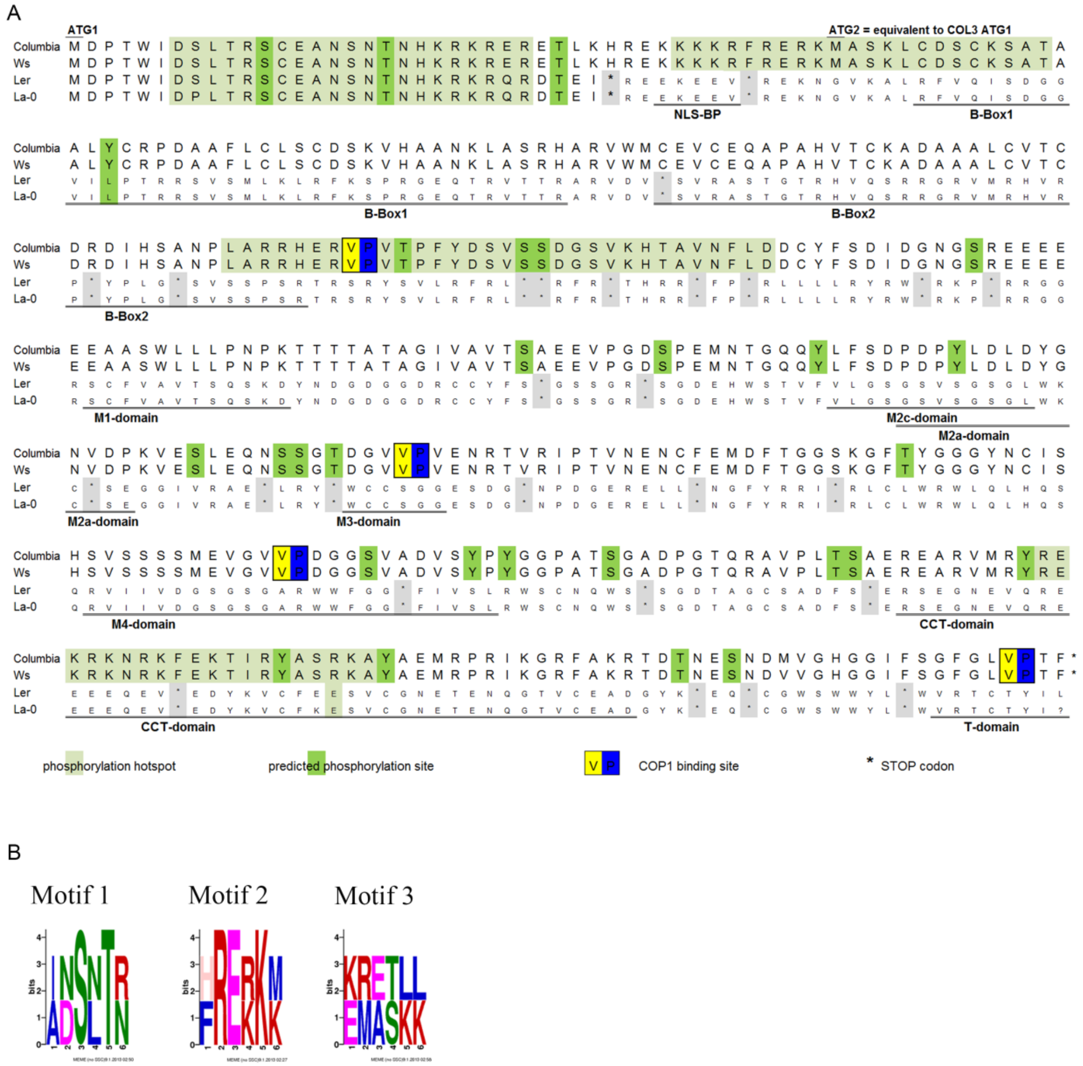

Fig.S 4: AtCOL4 Protein Sequence alignment of common ecotypes.

(A) Full Protein-sequence alignment of COL4 of the common ecotypes Columbia (Col), Wassilewskaja (Ws), Landsberg erecta (Ler) and Landsberg (La-0) using clustal W/ MEGA6 and BioEdit software. The sequence of Ler and La-0 harbors a premature stop codon at position 32 (G + A nucleotide deletion at position 82-83 of the genomic sequence) in the extra N-terminal sequence of COL4. Underlined amino-acids (AS) define the NLS-BP, the B-Box1/2, the M1-4-domains, the CCT-domain and the T-domain. (B) Motifs found in the N-terminal extension by using MEME (http://meme-suite.org/) such as INSNTR, KRFRERK, KMASKL, LKXXE.

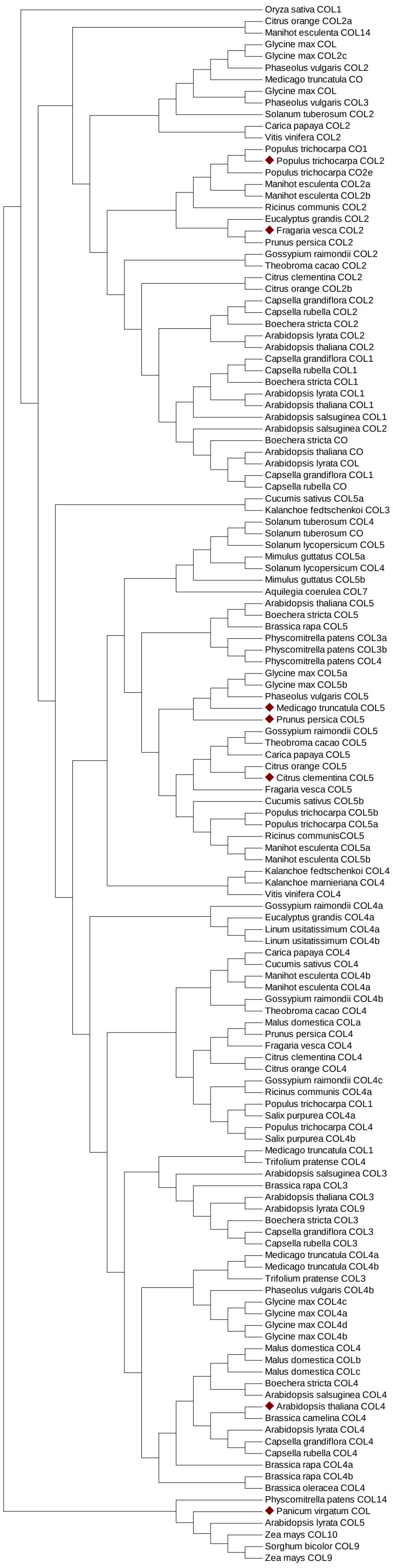

Fig.S 5: Molecular Phylogenetic analysis of COL4 like genes in green plant lineage.

The evolutionary history was inferred by using the Maximum Likelihood method based on the JTT matrix-based model [1]. The bootstrap consensus tree inferred from 500 replicates [2] is taken to represent the evolutionary history of the taxa analyzed [2]. Branches corresponding to partitions reproduced in less than 50% bootstrap replicates are collapsed. Initial tree(s) for the heuristic search were obtained by applying the Neighbor-Joining method to a matrix of pairwise distances estimated using a JTT model. The analysis involved 134 amino acid sequences. All positions with less than 95% site coverage were eliminated. That is, fewer than 5% alignment gaps, missing data, and ambiguous bases were allowed at any position. There were a total of 151 positions in the final dataset. Evolutionary analyses were conducted in MEGA6 [3].

1. Jones D.T., Taylor W.R., and Thornton J.M. (**1992**). The rapid generation of mutation data matrices from protein sequences. *Computer Applications in the Biosciences* **8**: 275-282.

2. Felsenstein J. (**1985**). Confidence limits on phylogenies: An approach using the bootstrap. *Evolution* **39**:783-791.

3. Tamura K., Stecher G., Peterson D., Filipski A., and Kumar S. (**2013**). MEGA6: Molecular Evolutionary Genetics Analysis version 6.0. *Molecular Biology and Evolution*30: 2725-2729.
