# Supplementary material for: The Arabidopsis thaliana CONSTANS-LIKE 4 (COL4) – A Modulator of Flowering Time
Source: Front Plant Sci. 2019 May 28;10:651. doi: 10.3389/fpls.2019.00651 (PMC6546890; doi:10.3389/fpls.2019.00651)
Supplement: Supplementary file 2 [file Data_Sheet_2.PDF]

Supplementary Material

The *Arabidopsis thaliana CONSTANS-LIKE 4* (*COL4*) – a modulator of flowering time

Yvonne Steinbach[1*]

*** Correspondence** to Yvonne Steinbach: yvonne.steinbach@uzh.ch

Supplementary Tables

Table S 1A: List of worldwide ecotypes from the 1001 ecotype set, carrying the common full length *COL4* gene encoding for functional COL4 protein. The analysis was performed with the genomes from the 1001 Genomes-project (The 1001 Genomes consortium 2016).

Table S 1B: **List of worldwide ecotypes** from the 1001 ecotype set, carrying the C-, GA- or GAGA-bp-deletion or other bp-changes in *COL4* causing premature stop codons and therefore resulting in non-functional COL4-protein. The analysis was performed with the genomes from the 1001 Genomes-project (The 1001 Genomes Consortium 2016).

Table S 2: **List of homolog protein sequences used for phylogenetic studies.**
Shown is the organism, Sequence ID, name in the phylogenetic tree, similarity to translational start site of: AtCOL4 (MASKL); AtCOL3 (MSSRL), AtCOL5 (MGFGLES), AtCOL2 (MLKEE).

Table S 3. Flowering time of Columbia *COL4* alleles grown in LD at DTB. Flowering time of *COL4* introgression lines (A) and *COL4*-oe lines in *col4*^{La} (B,C) or *co*-1 (D) mutant background in LD. Significance of difference was tested using t-tests. Asterisks denote differences that were significant at $p < 0.05$ to WT Col (*), WT La-0 (+), single mutant (**). Shown are mean value ± SE (n≥14).

Table S 4: Primers used for genotyping and cloning in this study.

Table S 5: Primers used for RT-qPCR and RT-sqPCR in this study. Shown are the forward and reverse primers with the appropriate Universal probe library (UPL) (Roche) probe number.

Table S 1A: List of worldwide ecotypes from the 1001 ecotype set, carrying the common full length *COL4* gene encoding for functional COL4 protein. The analysis was performed with the genomes from the 1001 Genomes-project (The 1001 Genomes consortium 2016).

ID	Name	origin	ID	Name	origin	ID	Name	origin
9503	11C1	UK	8256	Baa1-2	SWE	1062	Brösarp-15-138	SWE
8723	11PNA1,14	USA	9796	Bach2-1	GER	1063	Brösarp-21-140	SWE
8699	328PNA062	USA	9778	Bach-7	GER	1066	Brösarp-34-145	SWE
7566	627ME-13Y1	USA	9778	Bach-7	GER	1070	Brösarp-45-153	SWE
7568	627ME-1MI1	USA	9779	Bai-10	GER	531	BRR107	USA
7529	627RMX-1MN4	USA	9988	Bak-2	GEO	476	BRR12	USA
7530	627RMX-1MN5	USA	9121	Bak-5	GEO	484	BRR23	USA
7000	Aa-0	GER	9613	Balan-1	RUS	470	BRR4	USA
6986	Abd-0	UK	9332	Bar 1	SWE	504	BRR57	USA
9321	Ådal 1	SWE	9619	Basta-1	RUS	506	BRR60	USA
9323	Ådal 3	SWE	9620	Basta-2	RUS	7003	Bs-1	SUI
9323	Ådal 3	SWE	9621	Basta-3	RUS	7036	Bu-0	GER
9609	Adam-1	RUS	9935	BAU-15	FRA	7033	Buckhorn Pass	USA
6897	Ag-0	FRA	7028	Bch-1	GER	7058	Bur-0	IRL
9942	Agu-1	ESP	7013	Bd-0	GER	9830	Bus-0	ESP
9646	Aiell-1	ITA	9735	Bela-4	SVK	7062	Ca-0	GER
9939	Aitba-2	MAR	7008	Benk-1	NED	7061	Cal-0	UK
6987	Ak-1	GER	9775	Berg-1	GER	7063	Can-0	ESP
1166	Aledal-14-73	SWE	9824	Bes-5	ESP	9831	Cas-0	ESP
1158	Aledal-6-49	SWE	9928	BEZ-9	FRA	9679	Castelfed-1-195	ITA
991	Ale-Stenar-41-1	SWE	9813	BI-4	GER	9680	Castelfed-1-196	ITA
992	Ale-Stenar-44-4	SWE	9622	Bijisk-4	RUS	9681	Castelfed-1-197	ITA
997	Ale-Stenar-56-14	SWE	9761	Bik-1	LBN	9682	Castelfed-1-198	ITA
1006	Ale-Stenar-77-31	SWE	6900	Bil-5	SWE	9683	Castelfed-1-199	ITA
8230	Algutsrum	SWE	6901	Bil-7	SWE	9685	Castelfed-2-201	ITA
6989	Alst-1	UK	9648	Bisig-1	ITA	9686	Castelfed-2-202	ITA
9774	Alt-1	GER	7025	Bl-1	ITA	9687	Castelfed-2-203	ITA
9758	Altai-5	CHN	8264	Bla-1	ESP	9689	Castelfed-3-205	ITA
9970	Altenb-2	ITA	9825	Boa-0	ESP	9690	Castelfed-3-206	ITA
6990	Amel-1	NED	10004	Bolin-1	ROU	9691	Castelfed-3-207	ITA
9819	Amu-0	ESP	9336	Bön 1	SWE	9692	Castelfed-3-208	ITA
6898	An-1	BEL	5836	Boo2-3	SWE	9693	Castelfed-3-209	ITA
6992	Ang-0	BEL	7026	Boot-1	UK	9694	Castelfed-4-210	ITA
9980	Angel-1	ITA	9826	Bor-0	ESP	9695	Castelfed-4-211	ITA
9981	Angit-1	ITA	5837	Bor-1	CZE	9974	Castelfed-4-212	ITA
1313	Ängsö-59-422	SWE	6903	Bor-4	CZE	9975	Castelfed-4-213	ITA
1317	Ängsö-74-430	SWE	428	Borky1	CZE	9696	Castelfed-4-214	ITA
6680	Anholt-1	GER	9957	Borsk-2	RUS	9832	Cat-0	ESP
9759	Anz-0	IRN	9827	Bos-0	ESP	9937	CATS-6	FRA
9982	Apost-1	ITA	9339	Böt 1	SWE	9943	Cdm-0	ESP
5831	App1-14	SWE	9971	Bozen-1,1	ITA	7068	Cerv-1	ITA
5832	App1-16	SWE	9972	Bozen-1,2	ITA	9833	Cha-0	ESP
9820	Are-0	ESP	6904	Br-0	CZE	932	CHA-41	USA
9911	ARGE-1-15	FRA	9828	Bra-0	ESP	9624	Chaba-2	RUS
9927	ARR-17	FRA	5717	Bra-1	UK	7071	Chat-1	FRA
9821	Aru-0	ESP	9738	Bran-1	ROU	7072	Chi-0	RUS
7014	Ba-1	UK	9910	BRI-2	FRA	9705	Choto-1	BUL
8258	Bå4-1	SWE	1612	Brn-10	USA	6907	CIBC-17	UK
8259	Bå5-1	SWE	1622	Brn-24	USA	6908	CIBC-5	UK
7002	Baa-1	NED	8231	Brö1-6	SWE	9835	Cir-0	ESP

Follow up Table S 1A:

ID	Name	Origin	ID	Name	Origin	ID	Name	Origin
9912	CIRY-13	FRA	1651	DuckLkSP38	USA	6750	FM-11	USA
9983	Ciste-1	ITA	1652	DuckLkSP40	USA	9921	FOR-23	FRA
9984	Ciste-2	ITA	6008	Duk	CZE	7133	Fr-2	GER
7064	Cnt-1	UK	7107	Durh-1	UK	9381	Fri 1	SWE
7081	Co	POR	6009	Eden-1	SWE	9381	Fri 1	SWE
7077	Co-1	POR	6913	Eden-2	SWE	9382	Fri 2	SWE
6909	Col-0	USA	6010	Eden-5	SWE	9383	Fri 3	SWE
7092	Com-1	FRA	6011	Eden-6	SWE	9743	Furni-1	ROU
9837	Con-0	ESP	6012	Eden-7	SWE	6919	Ga-0	GER
10005	Copac-1	ROU	6013	Eden-9	SWE	9962	Galdo-1	ITA
9838	Cot-0	ESP	9298	Edi-1	UK	7161	Gd-1	GER
9839	Coy-0	ESP	9363	EdJ 2	SWE	8297	Ge-0	SUI
6744	CS28181	USA	6016	Eds-1	SWE	9125	Geg-14	ARM
7307	CS28645	FRA	6017	Eds-9	SWE	7143	Gel-1	NED
6739	CSHL-15	USA	9841	Ees-0	ESP	9909	GEN-8	FRA
6740	CSHL-17	USA	6915	Ei-2	GER	7147	Gie-0	GER
7067	Ct-1	ITA	9369	EkS 2	SWE	9653	Giffo-1	ITA
6911	Cvi-0	CPV	9370	EkS 3	SWE	9848	Glo-1	ESP
7460	Da(1)-12	CZE	7117	El-0	GER	9777	Gn-1	GER
7094	Da-0	GER	9843	Elp-0	ESP	9790	Gn2-3	GER
9840	Dar-0	ESP	7109	Ema-1	UK	9698	Goced-1	BUL
7419	Db-1	GER	8290	En-1	GER	6920	Got-22	GER
8233	Dem-4	USA	7119	En-2	GER	7158	Gr-5	AUT
7096	Di-G	FRA	7120	En-D	GER	7160	Gre-0	USA
9920	DIR-9	FRA	7125	Er-0	GER	9714	Grivo-1	BUL
766	Dja-1	KGZ	7126	Es-0	FIN	6025	Gro-3	SWE
7102	Do-0	GER	9908	ESP-1-11	FRA	9386	Grön 12	SWE
10018	Dobra-1	SRB	7127	Est	EST	9388	Grön 14	SWE
9353	Död 3	SWE	7130	Et-0	FRA	6030	Grön-5	SWE
9697	Dolen-1	BUL	9762	Etna-2	ITA	9849	Gud-3	ESP
9944	Don-0	ESP	9845	Evs-0	ESP	8234	Gul1-2	SWE
5856	Dör-10	SWE	9846	Ezc-2	ESP	8214	Gy-0	FRA
9706	Dospa-1	BUL	6917	Fäb-2	SWE	7461	H55	CZE
410	Doubravnik7	CZE	6918	Fäb-4	SWE	7163	Ha-0	GER
7106	Dr-0	GER	9371	FäL 1	SWE	9390	Had-1	SWE
7103	Dra-0	CZE	9941	Fei-0	POR	9391	Had-2	SWE
5865	Dra1-4	SWE	9847	Fel-2	ESP	9392	Had-3	SWE
5867	Dra2-1	SWE	9814	Fell1-10	GER	9394	Hag-2	SWE
5860	Dra-3	SWE	9776	Fell3-7	GER	9785	Ha-HBT1-2	GER
8283	Dra3-1	SWE	7138	Fi-0	GER	9797	Ha-HBT2-10	GER
424	Draha2	CZE	9651	Filet-1	ITA	9815	Ha-HBT3-11	GER
8284	DraII-1	CZE	8422	Fjä1-1	SWE	9395	Hal-1	SWE
5874	DraII-6	CZE	6019	Fjä1-2	SWE	9799	Hart-2	GER
8285	DraIII-1	CZE	6020	Fjä1-5	SWE	9791	Häs-1	GER
5893	DraIV 1-11	CZE	6021	Fjä2-4	SWE	9801	Ha-SP-2	GER
5890	DraIV 1-8	CZE	6022	Fjä2-6	SWE	7164	Hau-0	DEN
5907	DraIV 2-9	CZE	6023	Fly2-1	SWE	1684	Haz-10	USA
5921	DraIV 3-7	CZE	6024	Fly2-2	SWE	1676	Haz-2	USA
5950	DraIV 5-12	CZE	9380	FlyA 3	SWE	9769	HE-1	GER
5993	DraIV 6-22	CZE	6749	FM-10	USA	9850	Hec-0	ESP

Follow up Table S 1A:

ID	Name	Origin	ID	Name	Origin	ID	Name	Origin
7169	Hh-0	GER	9548	IP-Hoy-0	ESP	9701	Ivano-1	BUL
9787	HI-4	GER	9549	IP-Hum-2	ESP	9986	Jablo-1	BUL
9995	HKT2,4	GER	9550	IP-Iso-4	ESP	7181	Je-0	GER
7165	Hn-0	GER	9551	IP-Jim-1	ESP	10020	Jl-2	CZE
8235	Hod	CZE	9552	IP-Lab-7	ESP	7424	Jl-3	CZE
9772	Höf-1	GER	9553	IP-Ldd-0	ESP	7177	Jm-0	CZE
9404	Hola-1 1	SWE	9555	IP-Mar-1	ESP	9408	Kal-2	SWE
9405	Hola-1 2	SWE	9556	IP-Men-2	ESP	763	Kar-1	KGZ
9407	Hola-2 2	SWE	9557	IP-Moa-0	ESP	9617	Karag-1	RUS
6034	Hov1-7	SWE	9558	IP-Moc-11	ESP	9608	Karag-2	RUS
6036	Hov3-2	SWE	9559	IP-Mon-5	ESP	9708	Kardz-1	BUL
6038	Hov3-5	SWE	9508	IP-Mos-1	POR	9717	Kardz-2	BUL
8306	Hov4-1	SWE	9560	IP-Mot-0	ESP	7183	Kas-1	IND
6039	Hovdala-2	SWE	9561	IP-Mun-0	ESP	8424	Kas-2	IND
8307	Hovdala-6	SWE	9562	IP-Mur-0	ESP	10006	Kastel-1	UKR
6923	HR-10	UK	9564	IP-Nog-17	ESP	8237	Kävlinge-1	SWE
6924	HR-5	UK	9565	IP-Orb-10	ESP	7202	Kb-0	GER
7162	Hs-0	GER	9567	IP-Pal-0	ESP	9770	KBG2-13	GER
6805	HS-12	USA	9569	IP-Pds-1	ESP	1739	KBS-Mac-68	USA
6806	HS-17	USA	9571	IP-Pro-0	ESP	1741	KBS-Mac-74	USA
9851	Hue-3	ESP	9573	IP-Rds-0	ESP	8420	Kelsterbach-4	GER
9744	Iasi-1	ROU	9509	IP-Reg-0	POR	7475	KEN	USA
8311	In-0	AUT	9574	IP-Rel-0	ESP	8238	Kent	UK
9852	Ini-0	ESP	9576	IP-Rev-0	ESP	1756	Ker-4	USA
9513	IP-Adc-5	ESP	9577	IP-Ria-0	ESP	1757	Ker-5	USA
9517	IP-All-0	ESP	9578	IP-Sac-0	ESP	9409	Kia 1	SWE
9518	IP-Alm-0	ESP	9581	IP-Sdv-3	ESP	9960	Kidr-1	RUS
9506	IP-Alo-0	POR	9583	IP-Sne-0	ESP	7192	Kil-0	UK
9519	IP-Ang-0	ESP	9584	IP-Stp-0	ESP	6926	Kin-0	USA
9521	IP-Bar-1	ESP	9585	IP-Svi-0	ESP	9951	Kly-1	RUS
9522	IP-Bea-0	ESP	9586	IP-Tam-0	ESP	9952	Kly-4	RUS
9523	IP-Ben-0	ESP	9587	IP-Tdc-0	ESP	7186	Kn-0	LTU
9524	IP-Ber-0	ESP	9589	IP-Tor-1	ESP	6040	Kni-1	SWE
9526	IP-Cab-3	ESP	9591	IP-Vad-0	ESP	7717	KNO1,37	USA
9527	IP-Cad-0	ESP	9592	IP-Vae-2	ESP	6927	KNO-10	USA
9528	IP-Cal-0	ESP	9511	IP-Vav-0	POR	6814	KNO-15	USA
9529	IP-Cap-1	ESP	9593	IP-Vaz-0	ESP	7757	KNO2,41	USA
9530	IP-Car-1	ESP	9594	IP-Vdm-0	ESP	7767	KNO2,54	USA
9531	IP-Cdc-3	ESP	9595	IP-Vdt-0	ESP	7427	Ko-2	DEN
9533	IP-Cem-0	ESP	9596	IP-Ver-5	ESP	9699	Kolar-1	BUL
9534	IP-Cmo-3	ESP	9512	IP-Vid-1	POR	8239	Köln	GER
9507	IP-Coa-0	POR	9597	IP-Vig-1	ESP	9625	Kolyv-2	RUS
9535	IP-Coc-1	ESP	9598	IP-Vim-0	ESP	9626	Kolyv-3	RUS
9536	IP-Cor-0	ESP	9599	IP-Vin-0	ESP	9627	Kolyv-5	RUS
9537	IP-Cum-1	ESP	9600	IP-Vis-0	ESP	9628	Kolyv-6	RUS
9539	IP-Deh-1	ESP	9601	IP-Voz-0	ESP	6929	Kondara	TJK
9540	IP-Elb-0	ESP	9602	IP-Vpa-1	ESP	9412	Kor 3	SWE
9542	IP-Fun-0	ESP	9914	IST-29	FRA	9412	Kor 3	SWE
9544	IP-Gua-1	ESP	10012	Istisu-1	AZE	9413	Kor 4	SWE
9546	IP-Hom-4	ESP	9095	Istisu-5	AZE	9719	Koren-1	BUL
9547	IP-Hor-0	ESP	9099	Istisu-9	AZE	14312	Kos-1	RUS

Follow up Table S 1A:

ID	Name	Origin	ID	Name	Origin	ID	Name	Origin
14313	Kos-2	RUS	9085	Lerik2-7	AZE	9634	Masl-1	RUS
9953	Koz-2	RUS	9716	Leska-1	BUL	9864	Mat-0	ESP
9629	K-oze-1	RUS	9612	Lesno-2	RUS	9866	Mdd-0	ESP
9630	K-oze-3	RUS	9610	Lesno-4	RUS	1835	Mdn-10	USA
9616	Krazo-1	RUS	7223	Li-2:1	GER	1834	Mdn-8	USA
7203	Krot-0	GER	8241	Liarum	SWE	7250	Me-0	GER
9416	Kru-3	SWE	685	LI-EF-011	USA	9657	Melic-1	ITA
8240	Kulturen-1	SWE	687	LI-EF-018	USA	9703	Melni-1	BUL
9781	Kus2-2	GER	8242	Lillö-1	SWE	9704	Melni-2	BUL
9802	Kus3-1	GER	915	LIN S-5	USA	9946	Mer-6	ESP
801	KYC-33	USA	630	LI-OF-065	USA	7255	Mh-0	POL
7207	Kyoto	JPN	680	LI-RR-096	USA	853	MIA-1	USA
6830	Kz-13	KAZ	681	LI-RR-097	USA	854	MIA-5	USA
6931	Kz-9	KAZ	8222	Lis-2	SWE	867	MIC-20	USA
7209	La-0	POL	6041	Lis-3	SWE	868	MIC-24	USA
9853	Lac-0	ESP	728	LI-SET-019	USA	8337	Mir-0	ITA
9854	Laf-1	ESP	7236	Litva	LTU	9664	Mitterberg-1-179	ITA
9100	Lag1-2	GEO	544	LI-WP-039	USA	9665	Mitterberg-1-180	ITA
9102	Lag1-4	GEO	546	LI-WP-041	USA	9973	Mitterberg-1-181	ITA
9103	Lag1-5	GEO	6933	LL-0	ESP	9666	Mitterberg-1-182	ITA
9104	Lag1-6	GEO	7217	Lm-2	FRA	9667	Mitterberg-1-183	ITA
9105	Lag1-7	GEO	6042	Lom1-1	SWE	9668	Mitterberg-2-184	ITA
9106	Lag1-8	GEO	6043	Löv-1	SWE	9669	Mitterberg-2-185	ITA
9990	Lag2,2	GEO	6046	Löv-5	SWE	9670	Mitterberg-2-186	ITA
9115	Lag2-10	GEO	9858	Loz-0	ESP	9671	Mitterberg-3-187	ITA
9111	Lag2-4	GEO	7520	Lp2-2	CZE	9672	Mitterberg-3-188	ITA
9114	Lag2-7	GEO	7521	Lp2-6	CZE	9673	Mitterberg-3-189	ITA
9963	Lago-1	ITA	8464	LP3413,31	USA	9676	Mitterberg-4-192	ITA
1819	Lak-12	USA	8483	LP3413,53	USA	9677	Mitterberg-4-193	ITA
1820	Lak-13	USA	1797	L-R-10	USA	1925	MNF-Che-2	USA
9855	Lam-0	ESP	1793	L-R-5	USA	1942	MNF-Che-47	USA
9421	Lan 1	SWE	9859	Lro-0	ESP	2016	MNF-Pin-39	USA
7208	Lan-0	UK	8334	Lu-1	SWE	2017	MNF-Pin-40	USA
108	LDV-18	FRA	9782	Lu3-30	GER	1851	MNF-Pot-10	USA
139	LDV-46	FRA	9792	Lu4-2	GER	1852	MNF-Pot-15	USA
7218	Le-0	NED	9860	Lum-0	ESP	1853	MNF-Pot-21	USA
9631	Lebja-1	RUS	8335	Lund	SWE	1872	MNF-Pot-75	USA
9632	Lebja-2	RUS	9861	Mac-0	ESP	1890	MNF-Riv-21	USA
9633	Lebja-4	RUS	9862	Mad-0	ESP	9868	Moe-0	ESP
9930	LEC-25	FRA	9803	Mäh-2	GER	9966	Monte-1	ITA
9987	Lecho-1	BUL	9906	Mah-6	ESP	9870	Moz-0	ESP
9857	Leg-0	ESP	9720	Malak-1	BUL	6938	Ms-0	RUS
9945	Leo-1	ESP	9964	Mammo-1	ITA	2106	MSGA-10	USA
7213	Ler-0	GER	9965	Mammo-2	ITA	2108	MSGA-12	USA
6932	Ler-1	GER	2053	Map-35	USA	2081	MuskSP-68	USA
10013	Lerik1-3	AZE	2057	Map-42	USA	2091	MuskSP-83	USA
9075	Lerik1-4	AZE	2031	Map-8	USA	7248	Mv-0	USA
9078	Lerik1-7	AZE	159	MAR2-3	FRA	8343	Na-1	FRA
9079	Lerik2-1	AZE	9915	MAR-4-16	FRA	9089	Nar-3	AZE
9081	Lerik2-3	AZE	9655	Marce-1	ITA	9091	Nar-5	AZE
9084	Lerik2-6	AZE	9656	Marti-1	ITA	9427	Näs 2	SWE

Follow up Table S 1A:

ID	Name	Origin	ID	Name	Origin	ID	Name	Origin
7430	Nc-1	FRA	2212	Pent-46	USA	6077	Rev-3	SWE
8246	NC-6	USA	2191	Pent-7	USA	7316	Rhen-1	NED
9873	Ndc-0	ESP	9879	Per-0	ESP	9890	Rib-1	ESP
9993	Nemrut-1	TUR	8354	Per-1	RUS	2239	Riv-25	USA
772	Neo-6	TJK	7296	Petergof	RUS	2240	Riv-26	USA
6943	NFA-10	UK	10017	Petro-1	SRB	7471	Rld-1	UNK
6944	NFA-8	UK	9805	Pfn-10	GER	8132	RMX3,22	USA
9658	Nicas-1	ITA	9771	Pfn-N2,2-6	GER	8171	RMX4,118	USA
9996	Nie1-2	GER	8243	PHW-2	ITA	9027	RMX413,85	USA
7273	No-0	GER	8244	PHW-34	FRA	7319	Rome-1	ITA
6945	Nok-3	NED	7298	Pi-0	AUT	7320	Rou-0	FRA
9635	Nosov-1	RUS	9880	Pib-1	ESP	9976	Rovero-1	ITA
9636	Noveg-1	RUS	9881	Pie-0	ESP	7515	RRS-10	USA
9637	Noveg-2	RUS	18694	Pien	RUS	7514	RRS-7	USA
9638	Noveg-3	RUS	9882	Pil-0	ESP	7322	Rsch-4	RUS
7258	Nw-0	GER	9883	Piq-0	ESP	7323	Rubezhnoe-1	UKR
9433	Nyl 13	SWE	8357	Pla-0	ESP	9997	Rue3,1-31	GER
6064	Nyl-2	SWE	9924	PLY-20	FRA	9891	Sal-0	ESP
6069	Nyl-7	SWE	7523	Pna-17	USA	9892	Sam-0	ESP
7276	Ob-0	GER	7917	PNA3,10	USA	18696	Samm	RUS
9874	Oja-0	ESP	7947	PNA3,40	USA	8247	San-2	SWE
7280	Old-1	GER	9707	Podvi-1	BUL	8376	Sanna-2	SWE
9727	Olympia-2	GRC	7306	Pog-0	CAN	9918	SAUL-24	FRA
6070	Omn-1	SWE	9948	Pra-6	ESP	9721	Schip-1	BUL
6071	Omn-5	SWE	9885	Prd-0	ESP	9807	Schl-7	GER
6073	ÖMö1-7	SWE	7305	Pt-0	GER	6961	Se-0	ESP
15591	OOE1-1	AUT	8037	PT1,52	USA	7332	Seattle-0	USA
19949	OOE2-1	AUT	8057	PT1,85	USA	7333	Sei-0	ITA
19950	OOE2-2	AUT	6951	Pu2-23	CZE	9894	Sen-0	ESP
19951	OOE23	AUT	6956	Pu2-7	CZE	9643	Sever-1	RUS
15592	OOE3-1	AUT	6957	Pu2-8	CZE	7327	Sf-1	ESP
15593	OOE3-2	AUT	9888	Pva-1	ESP	7328	Sf-2	ESP
7282	Or-0	GER	265	PYL-6	FRA	9895	Sfb-6	ESP
6074	Ör-1	SWE	9764	Qar-8a	LBN	7344	Sg-1	GER
9741	Orast-1	ROU	9949	Qui-0	ESP	10015	Sha	AFG
8351	Ost-0	SWE	6958	Ra-0	FRA	9958	Shigu-1	RUS
7287	Ove-0	GER	9806	Rä-2	GER	9959	Shigu-2	RUS
9875	Ovi-1	ESP	9768	Rä4-16	GER	14318	Shu-1	RUS
7288	Oy-0	NOR	9917	RAD-21	FRA	14319	Shu-2	RUS
9876	Pad-0	ESP	14314	Radk-1	RUS	7337	Si-0	GER
9607	Panik-1	RUS	14315	Radk-2	RUS	9745	Sij 1/96	UZB
9639	Panke-1	RUS	7314	Ragl-1	UK	10008	Sij-1	UZB
9615	Parti-1	RUS	8365	Rak-2	CZE	10009	Sij-2	UZB
2159	Paw-13	USA	9640	Rakit-1	RUS	10010	Sij-4	UZB
2166	Paw-20	USA	9641	Rakit-2	RUS	9442	Sim-1	SWE
2171	Paw-26	USA	9642	Rakit-3	RUS	1552	Sku-30	SWE
9877	Pdl-0	ESP	8366	Rd-0	GER	9985	Slavi-1	BUL
9947	Ped-0	ESP	6959	Rennes-1	FRA	9723	Slavi-2	BUL
9878	Pee-0	ESP	6960	Rennes-11	FRA	2276	SLSP-31	USA
2202	Pent-23	USA	6076	Rev-2	SWE	2278	SLSP-35	USA

Follow up Table S 1A:

ID	Name	Origin	ID	Name	Origin	ID	Name	Origin
2286	SLSP-67	USA	6109	T510	SWE	6188	TDr-1	SWE
2285	SLSP-69	USA	6111	T530	SWE	6198	TDr-13	SWE
9718	Smolj-1	BUL	6113	T550	SWE	6201	TDr-16	SWE
9897	Smt-1	ESP	6114	T570	SWE	6203	TDr-18	SWE
6963	Sorbo	TJK	6115	T580	SWE	6189	TDr-2	SWE
7343	Sp-0	GER	6118	T610	SWE	6191	TDr-4	SWE
6085	Sparta-1	SWE	6119	T620	SWE	6192	TDr-5	SWE
9451	Spro 2	SWE	6122	T670	SWE	6194	TDr-8	SWE
9452	Spro 3	SWE	6123	T680	SWE	6209	TEDEN 02	SWE
6966	Sq-1	UK	6124	T690	SWE	6210	TEDEN 03	SWE
6967	Sq-8	UK	6125	T710	SWE	9736	Teiu-2	ROU
6086	Sr:3	SWE	6126	T720	SWE	6214	TFÄ 04	SWE
8386	Sr:5	SWE	6128	T740	SWE	6216	TFÄ 06	SWE
9754	Sredn-1	SRB	6131	T780	SWE	6217	TFÄ 07	SWE
8387	St-0	SWE	6132	T790	SWE	6218	TFÄ 08	SWE
9998	Star-8	GER	6133	T800	SWE	6220	TGR 01	SWE
9713	Stara-1	BUL	6134	T810	SWE	6221	TGR 02	SWE
9757	Staro-1	SRB	6136	T840	SWE	7353	Tha-1	NED
9756	Staro-2	SRB	6137	T850	SWE	8227	THÖ 03	SWE
9453	Ste 2	SWE	6138	T860	SWE	9968	Timpo-1	ITA
9454	Ste 3	SWE	6140	T880	SWE	7354	Ting-1	SWE
9455	Ste 4	SWE	6141	T890	SWE	6231	TNY 04	SWE
2317	Ste-40	USA	6142	T900	SWE	7356	Tol-0	USA
9956	Stepn-1	RUS	6145	T930	SWE	7358	Tol-2	USA
9955	Stepn-2	RUS	6148	T960	SWE	7359	Tol-3	USA
9728	Stiav-1	SVK	6149	T970	SWE	6235	TOM 01	SWE
9729	Stiav-2	SVK	6150	T980	SWE	6237	TOM 03	SWE
9731	Stiav-3	SVK	6151	T990	SWE	6238	TOM 04	SWE
10023	Strand-1	NOR	7349	Ta-0	CZE	6240	TOM 06	SWE
6088	Stu1-1	SWE	6153	TAA 03	SWE	6241	TOM 07	SWE
6087	Stu-2	SWE	6154	TAA 04	SWE	6242	Tomegap-2	SWE
7347	Stw-0	RUS	6163	TAA 14	SWE	1254	Tos-82-387	SWE
765	Sus-1	KGZ	6166	TAA 18	SWE	1257	Tos-95-393	SWE
6090	T1000	SWE	9794	Tä-B1-2	GER	6243	Tottarp-2	SWE
6091	T1010	SWE	7350	Tac-0	USA	350	TOU-A1-88	FRA
6092	T1020	SWE	6169	TÅD 01	SWE	351	TOU-A1-89	FRA
6094	T1040	SWE	6169	TÅD 01	SWE	6244	TRÄ 01	SWE
6095	T1050	SWE	6172	TÅD 04	SWE	9926	TRE-1	FRA
6096	T1060	SWE	6173	TÅD 05	SWE	9900	Tri-0	ESP
6097	T1070	SWE	6174	TÅD 06	SWE	6970	Ts-1	ESP
6098	T1080	SWE	9810	Tä-KS-7	GER	6971	Ts-5	ESP
6099	T1090	SWE	6177	TÄL 03	SWE	7372	Tscha-1	AUT
6100	T1110	SWE	6180	TÄL 07	SWE	7373	Tsu-0	JPN
6101	T1120	SWE	6968	Tamm-2	FIN	9999	TueSB30-3	GER
6102	T1130	SWE	6969	Tamm-27	FIN	10001	TueV-13	GER
6104	T1160	SWE	9783	Tä-PK-7	GER	10002	TueWal-2	GER
6105	T450	SWE	9899	Tau-0	ESP	9470	Tur-4	SWE
6106	T460	SWE	9812	Tä-W1	GER	6258	TV-10	SWE
6107	T470	SWE	9816	Tä-WH	GER	6268	TV-22	SWE
6108	T480	SWE	6184	TBÖ 01	SWE	6276	TV-30	SWE

Follow up Table S 1A:

ID	Name	Origin	ID	Name	Origin	ID	Name	Origin
6284	TV-38	SWE	9901	Urd-1	ESP	403	Zdarec3	CZE
6252	TV-4	SWE	9902	Usa-0	ESP	6984	Zdr-1	CZE
6255	TV-7	SWE	7382	Utrecht	NED	6424	ZdrI 1-23	CZE
6296	UduI 1-11	CZE	9903	Val-0	ESP	6445	ZdrI 2-21	CZE
6390	UduI 3-36	CZE	15560	Valm	RUS	6434	ZdrI 2-9	CZE
6396	UduI 4-9	CZE	9969	Valsi-1	ITA	9709	Zerev-1	BUL
7378	Uk-1	GER	7517	Vår2-6	SWE	9710	Zerev-1	BUL
10022	Uk-3	GER	9476	VårA 1	SWE	7417	Zu-0	SUI
10027	Uk-6	GER	9904	Vas-0	ESP	7418	Zu-1	SUI
5811	UKID107	UK	9991	Vash-1	GEO	9644	Zupan-1	CRO
5718	UKID11	UK	9058	Västervik	SWE	6413	Ull3-4	SWE
5822	UKID116	UK	9933	VED-10	FRA	9471	Ull-A-1	SWE
5720	UKID13	UK	9905	Ven-0	ESP	9312	Ullapool-8	UK
5726	UKID19	UK	7384	Ven-1	NED	6975	Uod-1	AUT
5741	UKID36	UK	9978	Vezzano-2,2	ITA			
5748	UKID43	UK	9950	Vie-0	ESP			
5757	UKID52	UK	7387	Vind-1	UK			
5768	UKID63	UK	7394	Wa-1	POL			
5772	UKID67	UK	7477	WAR	USA			
5776	UKID71	UK	9938	WAV-8	FRA			
5779	UKID74	UK	7404	Wc-1	GER			
5784	UKID79	UK	6979	Wei-0	SUI			
5798	UKID93	UK	9766	Westkar-4	KGZ			
5395	UKNW06-102	UK	8419	Wil-1	LTU			
5486	UKNW06-233	UK	7413	Wil-2	LTU			
5644	UKNW06-481	UK	7411	Wl-0	GER			
5651	UKNW06-488	UK	7396	Ws-0	RUS			
5023	UKSE06-118	UK	6981	Ws-2	RUS			
5165	UKSE06-362	UK	6982	Wt-5	GER			
5236	UKSE06-470	UK	7415	Wu-0	GER			
5249	UKSE06-491	UK	10014	Xan-1	AZE			
5253	UKSE06-500	UK	9067	Xan-3	AZE			
5279	UKSE06-541	UK	9069	Xan-5	AZE			
5349	UKSE06-639	UK	9070	Xan-6	AZE			
4807	UKSW06-207	UK	10011	Yeg-1	ARM			
4826	UKSW06-226	UK	9128	Yeg-2	ARM			
4840	UKSW06-240	UK	9130	Yeg-4	ARM			
4857	UKSW06-257	UK	9131	Yeg-5	ARM			
4884	UKSW06-285	UK	9133	Yeg-7	ARM			
4900	UKSW06-302	UK	9134	Yeg-8	ARM			
4939	UKSW06-341	UK	2370	Yng-4	USA			
4958	UKSW06-360	UK	2412	Yng-53	USA			
9737	Ulies-1	ROU	7416	Yo-0	USA			
8427	Ull2-13	SWE	9481	Yst-1	SWE			
6973	Ull2-3	SWE	9748	Zagub-1	SRB			
6974	Ull2-5	SWE	768	Zal-1	KGZ			

Table S 1B: **List of worldwide ecotypes** from the 1001 ecotype set, carrying the C-, GA- or GAGA-bp-deletion or other bp-changes in COL4 causing premature stop codons in the translation sequence and therefore resulting in non-functional COL4-protein. The analysis was performed with the genomes from the 1001 Genomes-project (The 1001 Genomes Consortium 2016).

GA-Deletion						C-Deletion		
ID	Name	Origin	ID	Name	Origin	ID	Name	Origin

430	Gr-1	AUT	9647	Basen-1	ITA	6976	Uod-7	AUT
9700	Dolna-1	BUL	9649	Bivio-1	ITA	9645	Gradi-1	CRO
9712	Dolna-1	BUL	9606	Aitba-1	MAR	8236	HSm	CZE
7383	Van-0	CAN	9510	IP-Rei-0	POR	9823	Bae-0	ESP
9568	IP-Pan-0	ESP	9611	Lesno-1	RUS	9793	Rä-N2	GER
9817	Ace-0	ESP	9747	Zabar-1	SRB	9800	Ha-S-B	GER
9834	Cho-0	ESP	9755	Vajug-1	SRB	9808	Tä-B2-3	GER
9836	Cod-0	ESP	6202	TDr-17	SWE	9811	Tä-NK-12	GER
9822	Aul-0	ESP	6112	T540	SWE	7031	Bsch-0	GER
9514	IP-Adm-0	ESP	1002	Ale-Stenar-64-24	SWE	8312	Is-0	GER
9515	IP-Ala-0	ESP	8369	Rev-1	SWE	7346	Ste-0	GER
9520	IP-Ara-4	ESP	8369	Rev-1	SWE	7199	Kl-5	GER
9525	IP-Bis-0	ESP	6035	Hov1-10	SWE	7231	Li-7	GER
9554	IP-Lso-0	ESP	9402	Hel-3	SWE	7268	Np-0	GER
9541	IP-Fue-2	ESP	9402	Hel-3	SWE	9979	Voeran-1	ITA
9543	IP-Gra-0	ESP	9450	Spro 1	SWE	6997	Appt-1	NED
9545	IP-Her-12	ESP	9436	Puk-1	SWE	9739	Toc-1	ROU
9590	IP-Trs-0	ESP	5104	UKSE06-252	UK	9730	Bela-1	SVK
9588	IP-Tol-7	ESP	5577	UKNW06-403	UK	9733	Bela-2	SVK
9582	IP-Ses-0	ESP	5353	UKNW06-003	UK	9399	Ham-1	SWE
9844	Esn-2	ESP	5210	UKSE06-432	UK	9437	Puk-2	SWE
9867	Mie-1	ESP	5276	UKSE06-533	UK	8249	Vimmerby	SWE
9869	Moj-0	ESP	4779	UKSW06-179	UK	8326	Lis-1	SWE
9856	Lch-0	ESP	5800	UKID96	UK	9343	Dja 1	SWE
9871	Nac-0	ESP	9314	Gol-2	UK	9352	Död 2	SWE
9898	Som-0	ESP	1829	Mdn-1	USA	6193	TDr-7	SWE
9886	Pru-0	ESP	1943	MNF-Che-49	USA	5830	App1-12	SWE
9932	NOZ-6	FRA	2141	MSGA-61	USA	6195	TDr-9	SWE
88	CYR	FRA	870	MIC-31	USA	9057	Vinslöv	SWE
9929	ISS-20	FRA	742	LI-SET-036	USA	7342	Su-0	UK
9113	Lag2-6	GEO	7377	Tul-0	USA	7111	Edi-0	UK
7244	Mnz-0	GER	7525	Rmx-A180	USA	8077	PT2,21	USA
6940	Mz-0	GER	1954	MNF-Jac-12	USA	628	LI-OF-061	USA
9784	Erg2-6	GER						
9780	Fell2-4	GER	**Nonsense Deletions**			**GAGA-Deletion**		
9786	Ha-P-13	GER	**ID**	**Name**	**Origin**	**ID**	**Name**	**Origin**
9795	Wank-2	GER	9711	Dolna-1	BUL	9579	IP-San-10	ESP
9789	Obh-13	GER	9722	Groch-1	BUL	9809	Tä-KB-6	GER
9798	Ha-P2-1	GER	5984	DraIV 6-13	CZE			
9804	Obe1-15	GER	9887	Pun-0	ESP	**C-GAGA-Deletion**		
9725	Epidauros-1	GRC	9925	RUM-20	FRA	**ID**	**Name**	**Origin**
9726	Faneronemi-3	GRC	6922	Gu-0	GER	9532	IP-Cdo-0	ESP
9684	Castelfed-2-200	ITA	9660	Sarno-1	ITA			
9663	Teano-1	ITA	1061	Brösarp-11-135	SWE	**C-GA-Deletion**		
9661	Cimin-1	ITA	8426	Ull1-1	SWE	**ID**	**Name**	**Origin**
9678	Mitterberg-4-194	ITA	5151	UKSE06-325	UK	9788	KBG1-14	GER
9659	Pigna-1	ITA				7516	Vår2-1	SWE

Table S 2: **List of homolog protein sequences used for phylogenetic studies.**
Shown is the organism, Sequence ID, name in the phylogenetic tree, Similarity to translational start site of: AtCOL4 (MASKL); AtCOL3 (MSSRL), AtCOL5 (MGFGLES), AtCOL2 (MLKEE).

Org	Organism	Phytozome ID	Name	Similar to
Aco	Aquilegia coerulea	Aquca_068_00006.1	AcoCOL7	MGFGLES
Aly	Arabidopsis lyrata	489353_AL6G36600.t1	AlyCOL4	MASKL
Aly	Arabidopsis lyrata	477472_AL3G11680.t1	AlyCOL2	MLKEE
Aly	Arabidopsis lyrata	488429_AL6G26710.t1	AlyCO	MLKEE
Aly	Arabidopsis lyrata	488432_AL6G26730.t1	AlyCOL1	MLKEE
Aly	Arabidopsis lyrata	481339_AL4G15780.t1	AlyCOL9	MSSRL
Aly	Arabidopsis lyrata	473005_AL1G41560.t1	AlyCOL5	
Tha	Arabidopsis salsuginea	Thhalv10004744m	ThaCOL4	MASKL
Tha	Arabidopsis salsuginea	Thhalv10013895m	ThaCOL1	MLKEE
Tha	Arabidopsis salsuginea	Thhalv10015486m	ThaCOL2	MLKEE
Tha	Arabidopsis salsuginea	Thhalv10000258m	ThaCOL3	MSSRL
At	Arabidopsis thaliana	At5G24930.1	AtCOL4	MASKL
At	Arabidopsis thaliana	AtCOL5.1	AtCOL5	MGFGLES
At	Arabidopsis thaliana	AtCO.1	AtCO	MLKEE
At	Arabidopsis thaliana	AtCOL1.1	AtCOL1	MLKEE
At	Arabidopsis thaliana	AtCOL2.1	AtCOL2	MLKEE
At	Arabidopsis thaliana	AtCOL3.1	AtCOL3	MSSRL
Bst	Boechera stricta	Bostr.5763s0023.1.p	BstCOL4	MASKL
Bst	Boechera stricta	Bostr.26833s0489.1.p	BstCOL5	MGFGLES
Bst	Boechera stricta	Bostr.1460s0153.1.p	BstCOL2	MLKEE
Bst	Boechera stricta	Bostr.2902s0319.1.p	BstCO	MLKEE
Bst	Boechera stricta	Bostr.2902s0320.1.p	BstCOL1	MLKEE
Bst	Boechera stricta	Bostr.8705s0012.1.p	BstCOL3	MSSRL
Bol	Brassica oleracea capitata	Bol036182/ PTHR31319:SF4	BolCOL4	MASKL
Bra	Brassica rapa FPsc	Brara.F02700.1.p	BraCOL4a	MASKL
Bra	Brassica rapa FPsc	Brara.I00559.1.p	BraCOL4b	MASKL
Bra	Brassica rapa FPsc	Brara.B01196.1.p	BraCOL5	MGFGLES
Bra	Brassica rapa FPsc	Brara.D01502.1.p	COL3	MSSRL
Csa	Camelina sativa	XP_010421344.1	not incl	
Cgr	Capsella grandiflora	Cagra.11928s0002.1.p	CgrCOL4	MASKL
Cgr	Capsella grandiflora	Cagra.1194s0065.1.p	CgrCOL2	MLKEE
Cgr	Capsella grandiflora	Cagra.6845s0028.1.p	CgrCOL	MLKEE
Cgr	Capsella grandiflora	Cagra.6845s0029.1.p	CgrCOL1	MLKEE
Cgr	Capsella grandiflora	Cagra.1004s0003.1.p	CgrCOL3	MSSRL
Cru	Capsella rubella	Carubv10001230m	CruCOL4	MASKL
Cru	Capsella rubella	Carubv10001192m	CruCO	MLKEE
Cru	Capsella rubella	Carubv10001279m	CruCOL1	MLKEE
Cru	Capsella rubella	Carubv10015501m	CruCOL2	MLKEE
Cru	Capsella rubella	Carubv10023746m	CruCOL3	MSSRL
Cpa	Carica papaya	evm.model.supercontig_1332.1	CpaCOL4	MASKL
Cpa	Carica papaya	evm.model.supercontig_95.76	CpaCOL5	MGFGLES
Cpa	Carica papaya	evm.model.supercontig_70.6	CpaCOL2	MLKEE
Ccl	Citrus clementina	Ciclev10008813m	CclCOL4	MASKL
Ccl	Citrus clementina	Ciclev10008366m	CclCOL5	MGFGLES
Ccl	Citrus clementina	Ciclev10012022m	CclCOL2	MLKEE

Csi	Citrus orange	orange1.1g019028m	CsiCOL4	MASKL
Csi	Citrus orange	orange1.1g017166m	CsiCOL5	MGFGLES
Csi	Citrus orange	orange1.1g017738m	CsiCOL2a	MLKEE
Csi	Citrus orange	orange1.1g017819m	CsiCOL2b	MLKEE
Csa	Cucumis sativus	Cucsa.163000.1	CsaCOL4	MASKL
Csa	Cucumis sativus	Cucsa.092190.1	CsaCOL5b	MGFGLES
Csa	Cucumis sativus	Cucsa.089490.1	CsaCOL5a	
Egr	Eucalyptus grandis	Eucgr.C01876.1	EgrCOL4a	MASKL
Egr	Eucalyptus grandis	Eucgr.C01876.2	EgrCOL4b	MASKL
Egr	Eucalyptus grandis	Eucgr.I02094.1	EgrCOL2	MLKEE
Fve	Fragaria vesca	mrna14981.1-v1.0-hybrid	FveCOL4	MASKL
Fve	Fragaria vesca	mrna27383.1-v1.0-hybrid	FveCOL5	MGFGLES
Fve	Fragaria vesca	mrna04172.1-v1.0-hybrid	FveCOL2	MLKEE
Gma	Glycine max	Glyma04G058900.1	GmaCOL4c	MASKL
Gma	Glycine max	Glyma04g06240.1	GmaCOL4a	MASKL
Gma	Glycine max	Glyma06G059600.1	GmaCOL4d	MASKL
Gma	Glycine max	Glyma06g06300.1	GmaCOL4b	MASKL
Gma	Glycine max	Glyma.13G093800.1	GmaCOL5a	MGFGLES
Gma	Glycine max	Glyma.17G066600.1	GmaCOL5b	MGFGLES
Gma	Glycine max	Glyma.08G255200.1.p	GmaCOL	MLKEE
Gma	Glycine max	Glyma.13G050300.1	GmaCOL	MLKEE
Gma	Glycine max	Glyma.18G278100.1	GmaCOL2c	MLKEE
Gra	Gossypium raimondii	Gorai.001G039500.1	GraCOL4a	MASKL
Gra	Gossypium raimondii	Gorai.009G065600.1	GraCOL4b	MASKL
Gra	Gossypium raimondii	Gorai.010G245200.1	GraCOL4c	MASKL
Gra	Gossypium raimondii	Gorai.004G102000.1	GraCOL5	MGFGLES
Gra	Gossypium raimondii	Gorai.008G059900.1	GraCOL2	MLKEE
Kfe	Kalanchoe fedtschenkoi	Kaladp0011s0227.1.p	KfeCOL3	MASKL
Kfe	Kalanchoe fedtschenkoi	Kaladp0029s0144.1.p	KfeCOL4	MASKL
Kfe	Kalanchoe marnieriana	Kalax.0205s0038.1.p	KlaCOL4	MASKL
Lus	Linum usitatissimum	Lus10020105	LusCOL4a	MASKL
Lus	Linum usitatissimum	Lus10026909	LusCOL4b	MASKL
Mdo	Malus domestica	MDP0000128581	MdoCOL4	MASKL
Mdo	Malus domestica	MDP0000202669	MdoCOLa	MASKL
Mdo	Malus domestica	MDP0000244238	MdoCOLb	MASKL
Mdo	Malus domestica	MDP0000901915	MdoCOLc	MASKL
Mes	Manihot esculenta	cassava4.1_011192m	MesCOL4a	MASKL
Mes	Manihot esculenta	cassava4.1_011725m	MesCOL4b	MASKL
Mes	Manihot esculenta	cassava4.1_010137m	MesCOL5a	MGFGLES
Mes	Manihot esculenta	cassava4.1_010378m	MesCOL5b	MGFGLES
Mes	Manihot esculenta	cassava4.1_009557m	MesCOL2a	MLKEE
Mes	Manihot esculenta	cassava4.1_009662m	MesCOL2b	MLKEE
Mes	Manihot esculenta	cassava4.1_025867m	MesCOL14	MLKEE
Mtr	Medicago truncatula	Medtr1g013450.1	MtrCOL1	MASKL
Mtr	Medicago truncatula	Medtr3g105710.1	MtrCOL4a	MASKL
Mtr	Medicago truncatula	Medtr4g034640.1	MtrCOL4b	MASKL
Mtr	Medicago truncatula	Medtr4g128930.1	MtrCOL5	MGFGLES
Mtr	Medicago truncatula	Medtr7g018170.1	MtrCO	MLKEE
Micpu	Micromonas pusilla	56149_Micromonas pusilla CCMP1545	MicpuC3v2	
Mgu	Mimulus guttatus	mgv1a008870m.g	MguCOL5a	MGFGLES

Mgu	Mimulus guttatus	mgv1a009654m.g	MguCOL5b	MGFGLES
Os	Oryza sativa japonica	LOC_Os09g06464.1	OsCOL1	MLKXE
Pvi	Panicum virgatum	Pavir.Ia03698.1.p	PviCOL	
Pvu	Phaseolus vulgaris	Phvul.009G085300.1	PvuCOL4b	MASKL
Pvu	Phaseolus vulgaris	Phvul.003G149000.1	PvuCOL5	MGFGLES
Pvu	Phaseolus vulgaris	Phvul.004G046600.1	PvuCOL3	MLKEE
Pvu	Phaseolus vulgaris	Phvul.008G022800.1	PvuCOL2	MLKEE
Ppa	Physcomitrella patens	Pp1s364_5V6.1	PpaCOL4	
Ppa	Physcomitrella patens	Pp1s371_27V6.1	PpaCOL3a	
Ppa	Physcomitrella patens	Pp1s97_104V6.1	PpaCOL14	
Ppa	Physcomitrella patens	Pp1s97_109V6.1	PpaCOL3b	
Ptr	Populus trichocarpa	Potri.006G267700.1	PtrCOL1	MASKL
Ptr	Populus trichocarpa	Potri.018G013800.1	PtrCOL4	MASKL
Ptr	Populus trichocarpa	Potri.006G173600.1	PtrCOL5b	MGFGLES
Ptr	Populus trichocarpa	Potri.T094400.1	PtrCOL5a	MGFGLES
Ptr	Populus trichocarpa	Potri.004G108300.1	PtrCO2	MLKEE
Ptr	Populus trichocarpa	Potri.017G107500.1	PtrCOL2e	MLKEE
Ptr	Populus trichocarpa	Potri.T016900.1	PtrCO1	
Ppe	Prunus persica	ppa008143m	PpeCOL4	MASKL
Ppe	Prunus persica	ppa007083m	PpeCOL5	MGFGLES
Ppe	Prunus persica	ppa007007m	PpeCOL2	MLKEE
Rco	Ricinus communis	29848.m004493	RcoCOL4a	MASKL
Rco	Ricinus communis	27506.m000051	RcoCOL5	MGFGLES
Rco	Ricinus communis	29496.m000135	RcoCOL2	MLKEE
Spu	Salix purpurea	SapurV1A.0184s0410.1.p	SpuCOL4a	MASKL
Spu	Salix purpurea	SapurV1A.0314s0280.1.p	SpuCOL4b	MASKL
Sly	Solanum lycopersicum	Solyc12g096500.1.1	SlyCOL5	MGFGLES
Sly	Solanum lycopersicum	Solyc08g006530.2.1	SlyCOL4	
Stu	Solanum tuberosum	PGSC0003DMP400051144	StuCOL4	MGFGLES
Stu	Solanum tuberosum	PGSC0003DMP400051145	StuCO	MGFGLES
Stu	Solanum tuberosum	PGSC0003DMP400017799	StuCOL2	MLKEE
Sbi	Sorghum bicolor	Sobic.004G256200.1.p	SbiCOL3	
Tca	Theobroma cacao	Thecc1EG036860t1	TcaCOL4	MASKL
Tca	Theobroma cacao	Thecc1EG038829t1	TcaCOL5	MGFGLES
Tca	Theobroma cacao	Thecc1EG019107t1	TcaCOL2	MLKEE
Trp	Trifolium pratense	Tp57577_TGAC_v2_gene38832	TrpCOL4	MASKL
Trp	Trifolium pratense	Tp57577_TGAC_v2_gene1834	TprCOL3	MSSRL
Vvi	Vitis vinifera	GSVIVT01036037001	VviCOL4	MASKL
Vvi	Vitis vinifera	GSVIVT01036499001	VviCOL2	MLKEE
Zma	Zea mays	GRMZM2G075562_T01	ZmaCOL9	
Zma	Zea mays	GRMZM2G075562_T02	ZmaCOL9	

Table S 3. Flowering time of Columbia *COL4* alleles in LD at DTB. Flowering time of *COL4* introgression lines (A) and COL4-oe lines in *col4*[La] (B,C) or *co-1* (D) mutant background in LD. Significance of difference was tested using t-tests. Asterisks denote differences that were significant at $p < 0.05$ to WT Col (*), WT La-0 (+), single mutant (**). Shown are mean value ± SE (n≥14).

		DTB (LD130µE)			
A	Col	32.1	±	0.2	
	Col col4[La]	31.0	±	0.6	
	COL4oe-21	35.9	±	0.7	*/**
	COL4oe-27	40.8	±	0.8	*/**
	COL4oe-16	45.9	±	0.9	*/**
	COL4oe-19	47.8	±	1.1	*/**
	COL4oe-26	37.2	±	0.6	*/**
	COL4oe-23	34.7	±	0.9	*/**
B	La-0	27.6	±	0.5	
	La *co-1*	39.5	±	0.9	+
	co-1 COL4oe-1	36.1	±	0.6	+/**
	co-1 COL4oe-2	40.0	±	0.8	+
	co-1 COL4oe-8	38.2	±	0.7	+

Table S 4: Primers used for genotyping and cloning in this study.

Gene	Forward primer (fw)	Reverse primer (rv)	T-DNA primer
CO/co-1	caccGGATCCATATGTTGAAACAAGAGAGTAACG	GTTGACTCCGGCACAACAC	Digest - BfaI
COL1/col1-1	GGGACAGAGAAGCTAGAGTC	GTGATGTGGAAAGTGTCACC	LBb1.3
COL3/col3-1	CAAGCTTTGCAAGGCACATACCACC	CACTAGTGATGCTCTCTATATCTCTCTC	LBb1.3
COL4/col4-1	caccATGGACCCCACATGGATAGac	AGGACCACCGTAAGGATACG	LBb1.3
COL4/col4-2	AGGACCACCGTAAGGATACG	CACCATGGACCCCACATGGATAGAC	LBb1.3
col4^{La}	caccATGGACCCCACATGGATAGac	AGGACCACCGTAAGGATACG	Digest - BfaI
COL9/col9-1	caccATGGGTTACATGTGTGACTTC	CAGTTTTCGAACTCATGAACG	LBb1.3
FT	GGTGGAGAAGACCTCAGGAA	GGTTGCTAGGACTTGGAACATC	
ft-10		TATAACAGGAAACCTAGTCCTGCTC	GK8409
SOC1/soc1-2	GGATCCATGGTGAGGGGCAAAACTC	CTGAAACATCTGATCAAAAGCTG	
	TTGGGTTCACGTAGTGGGCCATCG		
LBb1.3	ATTTTGCCGATTTCGGAAC		
LB3	TAGCATCTGAATTTCATAACCAATCTCGATACAC		
GK8409	ATATTGACCATCATACTCATTGC		
For cloning			
MDP-COL4 g/cDNA	caccATGGACCCCACATGGATAGac	(CTA)[1]-AAATGTAGGTACAAGTCCG	
MAS-COL4 g/cDNA	caccATGGCGTCAAAGCTCTGCGA	(CTA)[1]-AAATGTAGGTACAAGTCCG	
CO cDNA	caccGGATCCATATGTTGAAACAAGAGAGTAACG	[1]GAATGAAGGAACAATCCCATATC	

(cta)[1]- for fusion with C-terminal tags, the STOP-codon in brackets was left out
cacc – gateway adapter for TOPO-cloning
g/cDNA – genomic/cDNA

Table S 5: Primers used for RT-qPCR and RT-sqPCR in this study.
Shown are the forward and reverse primers with the appropriate Universal probe library (UPL) (Roche) probe number.

Gene	Forward primer	Reverse primer	UPL[1] probe no.
ACT2	TTCCGCTCTTTCTTTCCAAG	CCATTGTCACACACGATTGG	#102
AP1	TAGGGCTCAACAGGAGCAGT	CCACCCATGTTGAGAAAAGG	#53
ATC	CATATCCCCCAAAGCAGAAC	GTGTGTTGGAATGGGGAGAG	#117
COL4	CATCATCGTCGATGGAAGTGG	AGGACCACCGTAAGGATACG	#157
FD	GGCAGAAAATGCAAGACTCA	TCTTTTTGGGTTGCTGAATTG	#46
FT	GGTGGAGAAGACCTCAGGAA	GGTTGCTAGGACTTGGAACATC	#138
TSF	TGGAGGAGACGACTTCAGAAA	GCTTGGACTCGGCACATC	#138
PP2A[2]	GGAGAGTGACTTGGTTGAGCA	CATTCACCAGCTGAAAGTCG	#82
**For semiquantitative RT-PCR**			
GAPDH[2]	ATGGCTTCGGTTACTTTCTCTGTC	TTCTTGGCACCAGCTTCAAT	-
COL4[3]	caccATGGACCCCACATGGATAGac	CTAAAATGTAGGTACAAGTCCG	
COL4[4]	caccATGGCGTCAAAGCTCTGCGA	CTAAAATGTAGGTACAAGTCCG	-
COL4[5]	CATCATCGTCGATGGAAGTGG	CTAAAATGTAGGTACAAGTCCG	

1) UPL probe - Universal Probe Library probe; 2) Reference gene 1; 3) used for allele test if knockout for expression of *MDP-COL4 = 1COL4* 4) used for allele test if knockout for expression of *MAS-COL4 = 2COL4* 5) used for allele test if knockout for expression of 3COL4
